# Achieving quantitative reproducibility in label-free multisite DIA experiments through multirun alignment

**DOI:** 10.1038/s42003-023-05437-2

**Published:** 2023-10-30

**Authors:** Shubham Gupta, Justin C. Sing, Hannes L. Röst

**Affiliations:** 1 Terrence Donnelly Centre for Cellular & Biomolecular Research, University of Toronto, Toronto, ON Canada; 2 Department of Molecular Genetics, University of Toronto, Toronto, ON Canada; 3 Department of Computer Science, University of Toronto, Toronto, ON Canada

**Keywords:** Proteomics, Proteome informatics

## Abstract

DIA is a mainstream method for quantitative proteomics, but consistent quantification across multiple LC-MS/MS instruments remains a bottleneck in parallelizing data acquisition. One reason for this inconsistency and missing quantification is the retention time shift which current software does not adequately address for runs from multiple sites. We present multirun chromatogram alignment strategies to map peaks across columns, including the traditional reference-based Star method, and two novel approaches: MST and Progressive alignment. These reference-free strategies produce a quantitatively accurate data-matrix, even from heterogeneous multi-column studies. Progressive alignment also generates merged chromatograms from all runs which has not been previously achieved for LC-MS/MS data. First, we demonstrate the effectiveness of multirun alignment strategies on a gold-standard annotated dataset, resulting in a threefold reduction in quantitation error-rate compared to non-aligned DIA results. Subsequently, on a multi-species dataset that DIAlignR effectively controls the quantitative error rate, improves precision in protein measurements, and exhibits conservative peak alignment. We next show that the MST alignment reduces cross-site CV by 50% for highly abundant proteins when applied to a dataset from 11 different LC-MS/MS setups. Finally, the reanalysis of 949 plasma runs with multirun alignment revealed a more than 50% increase in insulin resistance (IR) and respiratory viral infection (RVI) proteins, identifying 11 and 13 proteins respectively, compared to prior analysis without it. The three strategies are implemented in our DIAlignR workflow (>2.3) and can be combined with linear, non-linear, or hybrid pairwise alignment.

## Abstract (web summary)

A new way to align DIA type proteomics data is described and shown that retention time alignment increases data completeness and decreases quantitative variance in multicenter synthetic datasets and also real-world patient datasets.

## Introduction

Data independent acquisition (DIA) is a popular method to probe the proteome landscape of a biological sample in liquid chromatography coupled to tandem mass-spectrometry (LC-MS/MS). It is shown to have superior reproducibility and better quantitative performance compared to other methods, such as shotgun proteomics due to its fixed MS/MS acquisition scheme and MS2 based quantification^1^. In clinical studies, it is often necessary to analyze a large number of samples to identify trends or to achieve enough statistical power in genetically diverse populations^2,3^. In such large-scale studies, it is often practically infeasible to acquire all runs under homogeneous conditions at the same time on a single instrument. Thus, being able to compare data across larger time frames of LC-MS/MS acquisition or across multiple instruments is becoming increasingly important for MS-based proteomics.

For such large-scale DIA studies, major sources of non-biological variation are sample preparation, retention time (RT) shifts, ionization and mass-spectrometer related artifacts. The latter two could be corrected, at a substantial overhead cost with spiked-in standards^4^ and technical replicates^5^. To correct for systemic RT variation current methods either use spiked-in iRT standards^6,7^ or high-scoring common identifications^8,9^ which generates a global fit. The alignment accuracy of these methods is poor at the scale of peak-width^10^, thus making peak-selection challenging in MS2 chromatograms which have dense peak-crowding around the peak of interest. Moreover, global methods intrinsically assume a constant peak-elution order across runs. However, this assumption falls apart in multi-column datasets due to analyte-specific local shifts that are more pronounced in complex matrices such as whole-cell lysate or plasma^10–12^. Hence, mapping peaks across multiple LC columns in DIA and targeted proteomics is still challenging leading to large-scale proteomics studies to often forgo cross-sample retention time alignment altogether^5,11,13^.

While peak scoring in DIA data uses sophisticated machine learning algorithms (LDA, XGBoost^14^, DIA-NN^15^, etc.) to control error-rate in peak-selection, these algorithms often assess the quality of a peak in isolation, without incorporating the local context of nearby signals in the same chromatogram (or in other LC-MS/MS runs) into account. Hence, if there are multiple suitable candidates in the extracted-ion chromatograms (XICs), these algorithms do not guarantee that the same analyte is consistently quantified across multiple LC-MS/MS injections. We hypothesize that with proper signal-mapping, these inconsistencies can be corrected, thus, reducing the error-rate further.

DIA experiments acquire the fragment-spectra of ionized species across all experiments, producing highly reproducible chromatograms that are distorted only due to experimental variation, sample composition and column history. We have previously described a pairwise alignment algorithm that uses these MS/MS chromatograms for alignment and is capable of removing non-linear chromatographic distortions in DIA proteomics data^10,16^. The alignment algorithm maps XICs (signals) using dynamic programming at the same time constraining the alignment with a global fit. Thus, this hybrid alignment is able to map the XICs across all runs irrespective of the instrument or site (Supplementary Note 1).

Here, we incorporate this hybrid pairwise alignment, termed as signal alignment, into a complete quantitative “DIAlignR workflow” which can perform multirun alignment and produces a quantitative data-matrix for downstream statistical analysis (Fig. S1). The workflow uses three strategies: Star, Minimum spanning tree (MST) and Progressive for multi-run alignment. In order to produce a better quantitative data-matrix, retention time mapping in conjunction with peak-scoring is employed for selecting correct peaks. In case of a missing peak, DIAlignR workflow recaptures a peak by integrating the signal within the aligned time boundaries. To benchmark, we compare peak-selection by this integrated workflow on manually annotated peaks against peaks selected from machine-learning based scoring only^8,16^.

Previous comparison with manual annotations was performed on a homogenous chromatographic data which does not capture variations that may arise in large-scale studies. Therefore, we next use multisite benchmarking data where technical replicates of a HEK293 cell lysate were shipped to 11 different sites, and each site acquired 21 replicate-runs forming a total of 229 proteome measurements^13^. This experiment allows us to study our algorithm under heterogeneous conditions on a multi-laboratory setup with different instruments and operators but with a known ground truth. On this challenging dataset, we compare the performance of DIAlignR with the current state-of-the-art method, TRIC, which uses global pairwise alignment of peak-groups using MST^8^. In addition, to assess performance in cases where proteins exhibit varying proportions between different conditions, we analyzed a multi-species dataset^17^ consisting of Yeast, Human plasma, and *E. coli*. Besides that, we also evaluate the three multirun strategies for site-specific and cross-site alignment.

Finally, we wanted to study whether our improvements in quantitation translate into better biological insight. We first assess the performance on a small-scale dataset^8^ of bacterial growth in human plasma. Subsequently, we re-interrogate 949 plasma runs acquired under heterogeneous conditions from a longitudinal prediabetic cohort. Beside the inherent biological variability of plasma samples, the data-acquisition process was complicated by switching the LC column and instrument maintenance during the acquisition^11^. Analyzing the dataset with DIAlignR not only increases the number of significant proteins by more than 50% compared to the unaligned data-matrix, but also discovers new proteins associated with insulin resistance (IR) and respiratory viral infection (RVI) response. Many of the known biomarkers found in our analysis were not reported in the original paper, partially due to the inability to fully align cross-column runs.

## Results

### Validation through manually annotated peaks

**Fig. 1:**
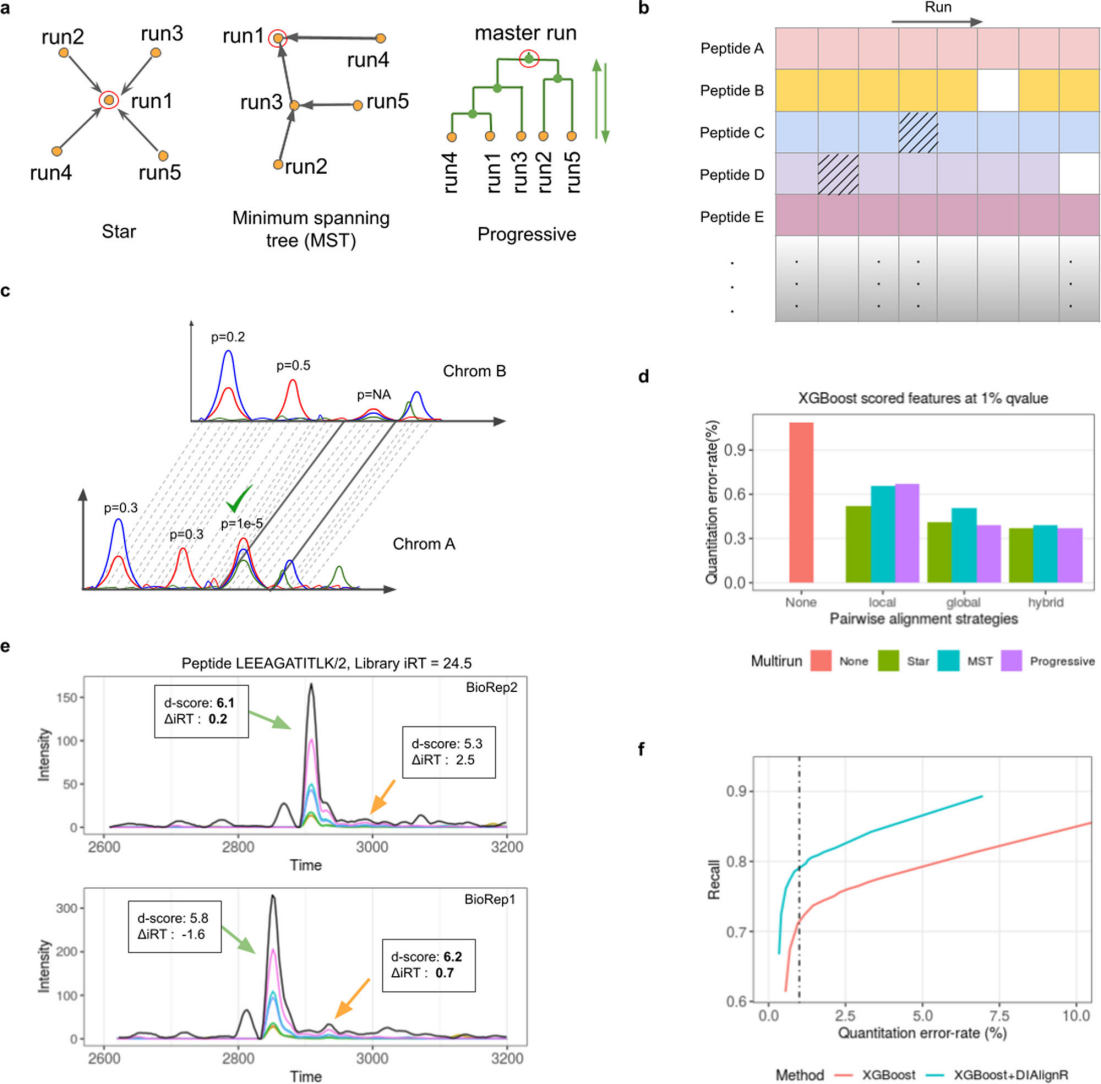
Benchmarking multirun alignment on a manually annotated dataset. **a** A schematic view of three multirun alignment strategies Star alignment, MST alignment and Progressive alignment. Yellow dots are LC-MS/MS runs, green dots indicate master runs created by merging two runs. Red circle indicates the seed run used for peak-mapping for a peptide. **b** An example peptide-intensity table produced from experiment-wide FDR control. Shaded cells indicate quantitation from incorrect-peaks, whereas blank cells indicate missing quantitation. **c** A high-quality feature is mapped from one run to another based on alignment. The generated feature would not have an associated *p* value. **d** The error-rate is calculated after comparing XGBoost and DIAlignR output with manual annotation. XGBoost *q* value cutoff is set to 1%. For DIAlignR, three pairwise alignment local, global and hybrid, and three multirun alignment strategies are explored. **e** Example chromatograms for a peptide from two runs. Black curve is library MS1 intensity, colored lines are MS/MS intensities. MS1 intensity is scaled by a factor of 0.4 for visualization. There are two confident peaks (*p* value < 0.01) found in each run. Peak selection, which relies on *d-*score, is inconsistent across both runs. *d-*score is in boldface for peaks selected by XGBoost. **f** Effect of alignment in complementing the machine learning scoring is demonstrated. More quantification events are reported at a constant error-rate with signal alignment.

There are three approaches implemented in DIAlignR to extend the pairwise alignment methods (Figs. 1a and S2–5):

Star alignment—for each peptide a seed run is selected to which pairwise alignment is performed for all runs, thus mapping the reference identification directly to other runs (Fig. S6a).

MST alignment—in this approach, the pairwise alignment is performed along a guide tree, hence, propagating the mapping from reference run to other runs.

Progressive alignment—in this approach, two runs are merged guided by a hierarchical tree (Fig. S6b). The process is followed until all runs are merged (Fig. S7), generating a master-run at the root. Thereafter, a reference peak is picked at the root, whose identification is mapped to downstream nodes while traversing back to the leaves.

In order to produce a peptide-intensity table, the alignment is followed by peak-selection with the aim to reduce both incorrect and missing quantitative events (Fig. 1b, c). Briefly, for the peptide quantification we select a peak with the lowest *p* value within a window about the aligned time. The algorithmic details of multirun methods and peak-selection are in Supplementary Note 2 and 3. Next, for some signals that are not picked by the initial peak-picker or excluded by subsequent scoring, we create new peaks by mapping the retention time boundaries; this is termed as signal integration (Fig. 1c). The signal between peak-boundaries is quantified using the OpenMS peak-integrator to keep parity with the upstream analysis. Besides quantifying missing peptides between runs, signal integration is also carried out within a run for multiple charge-states of a peptide where peak boundaries are transposed from the high-scoring charge-state to low-scoring one.

In addition to the multirun approaches, we are also interested in their combination with previously described pairwise alignment^10^. Three methods are discussed: (1) *global* which uses high-confident MS2 features to calculate either a linear or lowess fit, (2) *local* that uses MS2 XICs for each peptide, calculates a similarity matrix and finds the alignment path using dynamic programming, and (3) *hybrid* approach that constrains the similarity matrix with global fit before performing dynamic programming, thus combining best of both *local* and *global* methods. To validate and benchmark the peak-selection from pairwise and multirun alignment algorithms, we use a gold-standard data consisting of 16 lysate runs of *S. Pyogenes* grown in two conditions, without and with plasma (Supplementary Table 1). These cell lysates were acquired on a single column within 2 days^7^. There are manual annotations available for 437 randomly picked peptides which are used to calculate error-rate (see “Methods” and Supplementary Note 5). On this gold-standard data, our DIAlignR workflow reduces the number of incorrect peaks by more than 60% (from 56 to 19 incorrect) compared to without alignment (Fig. 1d). Out of three pairwise methods, the *hybrid alignment* performs the best, which is consistent with our previous study^10^. Hence, we have employed the hybrid pairwise alignment for the subsequent analyses. On the other hand, the resulting error-rate from the three multirun strategies is found to be equivalent with the hybrid approach. An example of inconsistent peak-selection is depicted in Fig. 1e, where the chromatograms for a peptide are extracted from two of the runs. The XICs have multiple potential peaks from which PyProphet, a peak-scorer, is unable to select consistently across runs due to slight RT deviations and resulting ΔiRT scores^14^. DIAlignR removes such inconsistencies and selects the correct peak. In addition to benchmarking against unaligned data-matrix, we have also used the manual annotations to optimize the parameters for MST and Progressive alignment including guide tree construction and merging of runs (Supplementary Note 4 and Figs. S8–13).

Increasing the stringency of the quantitative error-rate control can increase the accuracy but comes at the loss of quantification events. Here, we argue that cross-run alignment can improve the data-matrix accuracy without a subsequent loss in the number of quantified events while providing constant error rates (Fig. S6d). At 1% error-rate, only 72% of the data-matrix had peptide quantification events without any alignment. With the DIAlignR workflow, we increase the completeness by 10 to 79% without increasing the error-rate (Fig. 1f). As illustrated, the gain is consistent at lower and higher error-rates. Incorporating the peaks received from signal-integration further increased the completeness of the matrix by 2.5 percentage points to 81.5% at the same error-rate (Fig. S6c and Supplementary Note 5.2). Most peak pickers fail when the signal is very close to noise, hence miss such low intensity features^8,9,18^, whereas in other cases poor scores are assigned to correct peaks (Figs. S14–16). Signal integration based on RT mapping aims to correct such instances. We further investigated increasing the matrix completeness, achieving 98% completeness when slightly relaxing our criteria for quantitation error-rate to 5% while keeping the overall peptide-level FDR at 1%, thus avoiding imputation almost completely (Figs. S14a and S17).

### Benchmarking on multi-species data

Next, we analyzed a multi-species benchmarking dataset comprising Yeast, Human plasma, and *E. coli*. We assessed the performance of DIAlignR in cases where certain proteins are at different proportions in one condition compared to another. This evaluation allowed us to assess DIAlignR’s control of the quantitative error rate and investigate the effect of peak selection on a larger set of molecules. By comparing the aligned results to the unaligned results, we observed a reduction in the coefficient of variation (CV) of protein intensities for *E. coli*, Human, and Yeast. The aligned data exhibited reduced CV, indicating enhanced precision and decreased variability in the protein measurements, with CV decreases of ~3.57% for *E. coli*, 1.69% for Human, and 1.63% for Yeast.

**Fig. 2:**
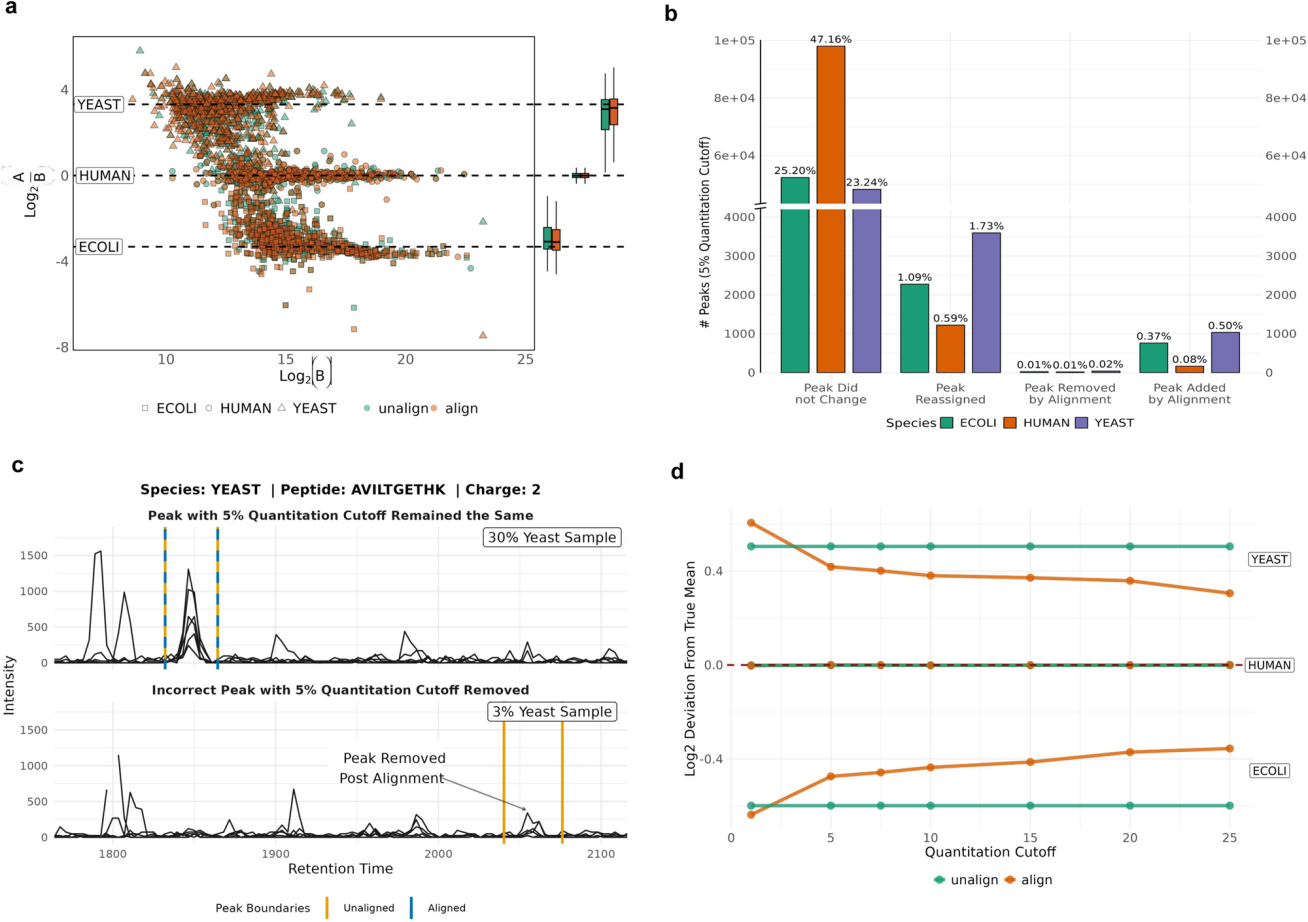
Evaluating quantitative error rate control on multi-species dataset. **a** Log-transformed ratios (log_2_(A/B)) of proteins over log-transformed intensities of Sample B for unaligned results (green) and aligned results (orange). Dashed black lines indicate the expected log_2_(A/B) ratios. **b** Number of quantitative peaks at 5% quantitation cutoff, separated into quantitation event changes incurred by alignment, and further broken into species specific proteins. **c** An example of two extracted ion chromatograms (XIC) for a yeast peptide. The top XIC is for a run of sample A containing 30% of yeast in the sample composition, and the bottom XIC is for a run of sample B containing 3% of yeast in the sample composition. Peak boundaries in orange represent the unaligned peak boundaries at 5% quantitation cutoff, and the blue dashed peak boundary represents the aligned peak boundary. **d** Deviation of the mean log_2_(A/B) ratios to the true expected mean ratios per species. The red dashed line represents the deviation of zero to the true mean. Labels for yeast, human and *E. coli* correspond to the pairs of unaligned and aligned lines.

Furthermore, at a 5% quantitative FDR, DIAlignR improved the precision of expected species ratios by reducing the variance of log ratios by 17% for *E. coli*, 6% for Human, and 8% for Yeast (Fig. 2a). Moreover, DIAlignR reassigns 3.41% of precursors based on aligned features. It also removes 0.04% of peaks when a suitable aligned feature is not found, while introducing 0.94% of new peaks that were not originally identified by the peak-picking algorithm (see Fig. 2b). Figure 2c illustrates an example where DIAlignR avoids aggressively adding peaks in the absence of suitable peak. In Run 001, which comprises 30% yeast species in the sample mix, a peak is present for the yeast peptide AVILTGETHK. However, no peak is observed for the 3% yeast composition samples in Run 006. By comparing unaligned and aligned results at different quantitation FDR cutoffs, we observed that DIAlignR aligns peaks conservatively, approaching the expected log ratio with tighter variances, particularly at higher quantitation FDR thresholds (Fig. 2d).

### Benchmarking on multisite technical data

To evaluate the alignment strategy across a heterogeneous dataset we have analyzed the data from Collins et al. measured on eleven different MS instruments at different geographic locations^13^. Without alignment, we quantify 52,529 peptide-ions from 4703 proteins at constant FDR of 1% for both peptide and protein, similar to the analysis presented in the original study (Supplementary Note 6.3, Supplementary Table 2 and Fig. S18). As the same samples were measured, we employ the coefficient of variation (CV) of analyte intensity across all runs to measure the alignment quality. The intensities are normalized with the coefficients from the original study to avoid its effect on CV.

**Fig. 3:**
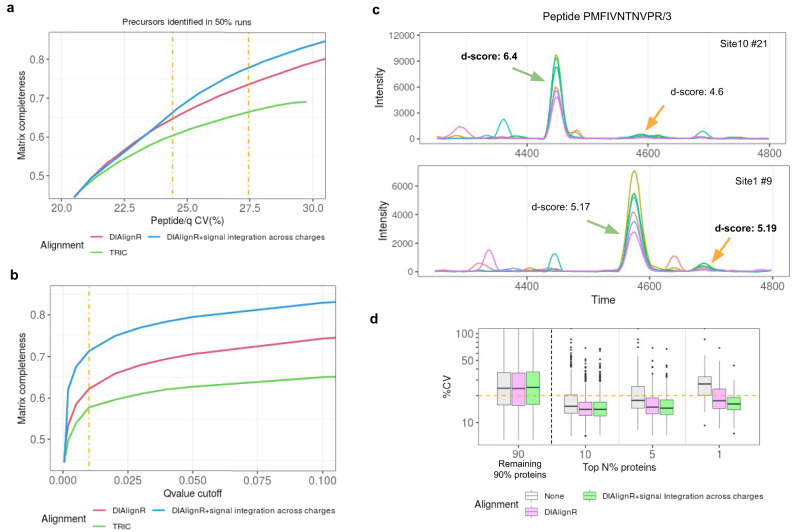
Comparison of signal alignment by DIAlignR on multisite data. To measure completeness 54,492 analytes are considered which are quantified in at least half of the runs. **a** Effect of matrix completeness on CV is presented for TRIC and DIAlignR without and with signal integration across charges (considered for *q* value > 0.001). Dot-dash line represents 0.01 and 0.05 cut-offs with CV being 24 and 27%. **b** Effect of PyProphet *q* value cut-off on matrix completeness. **c** Example chromatograms for a peptide from two sites with colored lines representing MS/MS signal. Peak selection, which relies on *d-*score, is inconsistent across both runs. *d-*score is in boldface for peaks selected by pyProphet. **d** CV of high intensity proteins (*n* = 4604) is depicted without and with signal alignment.

First, we compare DIAlignR workflow to the TRIC software^8^ using a minimum spanning tree (MST) approach for mapping retention time across runs for both methods. The quantitative data-matrix from TRIC has a higher CV for the same recall when compared to DIAlignR, irrespective of signal integrated peaks (Fig. 3a). This is because the global fit, used by TRIC, alone fails to map peaks across multiple columns, as demonstrated in ref. ^10^. The hybrid alignment algorithm does not suffer from the local retention time shifts and can map peaks successfully across columns, thus DIAlignR produces 10% more quantification events which translates as 3224 more quantified ions per run at constant 24% CV (Fig. 3b). Since many true peaks were removed by TRIC due to incorrect cross-column alignment, the produced data-matrix never reaches more than 70% completeness. DIAlignR workflow instead removes spurious peaks, at the same time improved RT mapping allows more quantitative events to be pulled in. This gives an opportunity to re-analyze some of the large-scale multi-column studies^5,11,13^. Our results are robust across a large range of commonly used error-rate cut-offs between 0.01 to 10%, where DIAlignR has superior performance for data-matrix completeness compared to TRIC. Analysis with signal integration across runs produces results similar to signal integration across charges (Fig. S19a, b), especially above 5% quantitative error-rate.

Subsequently, we were interested-in whether the additional peaks picked by our algorithm deteriorate CV. Surprisingly, we find not only that CV stayed consistent even when increasing matrix completeness, but also improved in certain cases, especially for high abundant ions. One of these cases is shown in Fig. 3c, where the chromatograms of a peptide are shown from two different sites. The peak classifier picks different peaks in both runs based on an aggregate discriminant score (Fig. S19c). For this data, the classifier calculates a higher weight for the transition-intensity correlation instead of ΔiRT. In conjunction with classifier scores, DIAlignR workflow also uses RT mapping, thus selects correct peaks successfully in both runs. This phenomenon also translates to the protein level, as there is about 15% drop in CV for the 5% most intense proteins out of 4604 quantified (Fig. 3d). Although modest, quantitative reproducibility also improves for low abundant proteins. DIAlignR picks correct peaks consistently from a pool of potential good candidates for intense proteins, whereas new peaks are generated for less abundant ones (Fig. S20).

### Comparison of multirun alignment strategies

**Fig. 4:**
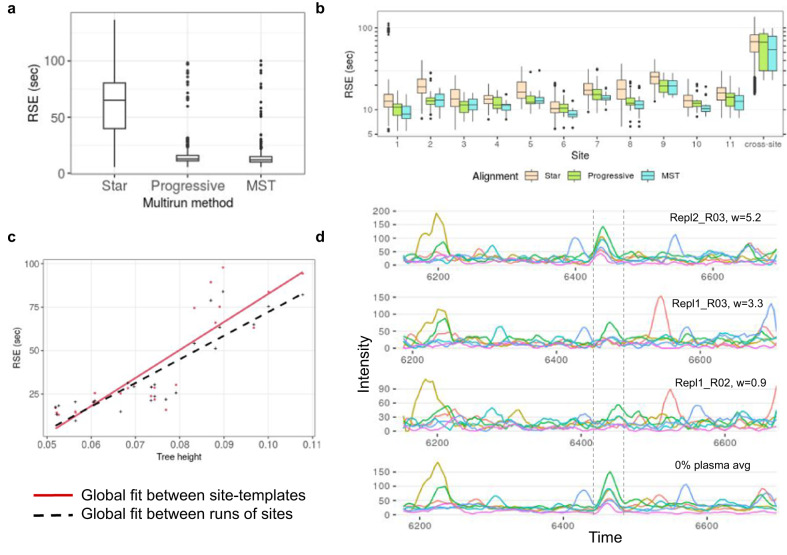
Comparison of Star, MST and Progressive alignments. **a** RSEs of global fits used in the Star, Progressive, and MST alignment of 229 runs. **b** RSEs of global fits used in the intra-site alignments and cross-site alignments. **c** Mean RSE from the Progressive alignment of site-specific templates v/s mean RSE of global fits between runs of these sites. **d** An example of aligned chromatograms of peptide TVASNPQGFFDILMAPVR/3 from *S. Pyogenes* cell lysate data. The six runs are weighted averaged during progressive alignment to produce a merged chromatogram, shown at the bottom. Only three runs out of six are depicted here. The correct peaks are overlapping the dashed boundary from the merged chromatogram.

Next, we were curious if any difference could be discerned between Star, MST, and Progressive alignment on this heterogeneous dataset. For both site-specific and cross-site alignments, all three methods reduce CV compared to no alignment (Table 1). Nonetheless, the MST approach produces the lowest CV for both cross-site and site-specific alignment. The hybrid pairwise method uses global fit to constrain the alignment path; a global fit is usually a non-linear RT mapping generated from confident common peaks in a run-pair. We notice that the global fits from the Star approach have unusually higher residual standard error (RSE) than other multirun methods (Fig. 4a), on top of that, it also requires many more global fits (Supplementary Table 4). MST and Progressive alignments, in contrast, employ fewer global fits, and by design only neighboring runs are selected to build the tree. Therefore, the generated global fits have comparatively lower RSE irrespective of site as depicted in Fig. 4b. Moreover, Star alignment is susceptible to a poor global fit as some pairs may not have enough high-scoring common features. This situation is avoided in Progressive and MST alignment where such scenarios are factored-in for tree-construction.

**Table 1:** Comparison of multirun methods at 1% *q* value for 34,202 precursors.

Multirun method	CV %		Global fit RSE (sec)	
	Cross-site (229 runs)	Site-specific (11 sites)	Cross-site only	Site-specific only
None	24.0 ± 11.8	14.5 ± 12.1		
Star	23.5 ± 11.2	13.9 ± 12.2	68 ± 23.2	15.3 ± 9.3
Progressive	23.6 ± 10.7	13.8 ± 11.1	67 ± 27.7	12.4 ± 4.7
**MST**	**23.4** ± **10.3**	**13.8** ± **11.0**	**54** ± **26.8**	**11.7** ± **3.9**

Minimum CV and RSE are in boldface.

Although the reference-free Progressive method provides site-specific CV equivalent to MST, surprisingly, it performs poorer than the Star method for cross-site alignment. This is attributed to the methodology behind Progressive alignment where runs are merged assuming homogeneity in chromatography. As the tree is traversed the RSE of corresponding global fits increase due to the amalgamation of distant runs. However, in the case of cross-site template merging, the produced global fit is more distant than what would be expected from the alignment of leaf-runs, as happens in Star alignment (Fig. 4c). Thus, the successive merging of individual geographic sites’ templates struggles to assimilate the chromatographic heterogeneity between sites, whereas such occurrences are uncommon in site-specific merging (Fig. S21a).

Merging chromatographic traces has been shown for GC-MS data; however to our knowledge, it has not been attempted for much more complex HPLC data, let alone SWATH/DIA-MS. The progressive alignment strategy generates a master chromatogram which is a weighted average of all parent XICs. In Fig. 4d, we demonstrate an example of such chromatogram-averaging. Due to averaging, noise is reduced and signal is enhanced and thus the merged XICs appear smoother than individual traces. Besides visualization, the recurring signals are also captured in the template XICs.

### Bacterial growth in plasma with DIAlignR

We, next, analyzed the effect of plasma on the growth of *S. Pyogenes* both without and with alignment (Supplementary Note 7). The differential proteomics analysis resulted in 67 statistically significant proteins out of 1001, increasing the number of differential proteins from 60 without alignment. The additional proteins we identify are not due to the increased *q* value cut-off (Supplementary Table 6), instead are ensued from improved peak selection by alignment that increased statistical confidence during the differential analysis (Supplementary Table 7). One of the newly identified proteins is *hasB* (Figs. S22–27) which is a known virulence factor and is present on the same operon as another significant hit, *hasA*. The progressive alignment also generates a single chromatogram per condition, enabling a single chromatographic visualization from multiple runs (Supplementary Note 7.4). The example chromatograms of *hasB* protein are displayed in Fig. S21b.

### Reanalyzing prediabetic cohort with DIAlignR

Encouraged by the technical analysis, we next explored how the improvement by DIAlignR translates into clinical insights by re-analyzing 949 plasma runs from a cohort of 107 prediabetic participants^11,19^. Briefly, samples were collected quarterly when participants self-reported as healthy for up to 8 years (Supplementary Note 8). Additional visits occurred during the periods of respiratory viral infection. All 949 runs were aligned and processed as described in “Methods”.

**Fig. 5:**
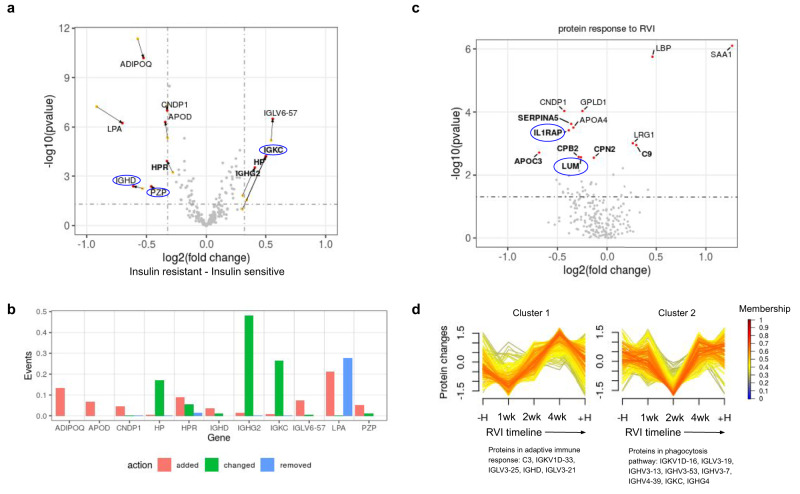
Analysis with aligning 949 plasma runs. **a** Volcano plot depicting significant proteins from the differential analysis as red dots, and their values from pre-aligned analysis are depicted as yellow dots. Genes that are called significant after the alignment are in boldface. Proteins with no-literature association to insulin sensitivity are in blue oval. **b** The effect of DIAlignR on quantitation events for genes associated with IR. **c** Volcano plot depicting 13 proteins that change significantly during RVI. Proteins that are called significant after alignment are in boldface. The fold change is from 1 week after infection—healthy baseline. **d** Two temporal clusters that are altered with aligned data. The core proteins, based on membership values, are mentioned below each cluster.

Firstly, we analyze healthy baseline samples (*n* = 416) to identify proteins that differ among insulin-sensitive (IS) to insulin-resistant (IR) subjects. Linear mixed-effect model is employed for differential proteome analysis with batch, participant ID and acquisition order as random effects. With aligned data, we identify 11 associated proteins (Fig. 5a); adding four proteins HP, HPR, IGKC and IGHG2 to the initial results without alignment, translates to 57% increment in the number of associated proteins. DIAlignR picks the correct peak for these proteins which lead to increased quantification events and tighter variance estimates, resulting in stronger *p* values (Supplementary Tables 8 and 9). However, this is not the case for all proteins; the *p* value increased for ADIPOQ with quantification of more events, whereas for LPA a different peptide is picked for analysis from the aligned data-matrix (Fig. 5b). To visualize the differential protein abundance, we removed batch, acquisition-order, and participant-specific effects from each sample. The resulting distribution is presented in Fig. S28a.

Besides recapturing known biomarkers for insulin resistance, we have been able to discover three novel proteins, to the best of our knowledge, associated with insulin resistance: IGHD, IGKC and PZP. While the remaining eight proteins are known in the literature, surprisingly many of these were not reported in the original analysis (Supplementary Note 8.5). Below we summarize the role of biomarkers for which literary evidence is available.

#### Biological significance of differential proteins

Out of the four new identifications by DIAlignR, three proteins (HPR, HP, and IGHG2) are known to be associated with IR. Haptoglobin-related protein (HPR) forms a subclass of apoL-I containing HDLs whose levels are negatively correlated with insulin resistance in humans^20^. In contrast, Haptoglobin (HP) was not known to be associated with IR in humans, but is highly consistent with animal studies where HP-null mice showed protection against insulin resistance^21^. Our results contain two new immune system related proteins (IGKC and IGHD) which are consistent with other two known immunological proteins (IGHG2 and IGLV6-57) associated with insulin sensitivity. The IGHG2 gene encodes the C-region of gamma-2 heavy chain that defines IgG2 isotype whose pathogenic role was previously reported to be connected to insulin resistance in a cross-sectional study of 262 participants^22^. IGLV6-57 encodes the variable domain of immunoglobulin light chains and has been associated with diabetes mellitus previously^23^. Out of the three novel proteins, immunoglobulin kappa constant gene (IGKC) was reported to be overexpressed in low-responders with high HOMA-IR during a diet intervention study for obese boys^24^. The pregnancy zone protein (PZP) has not directly been associated with IR, although recently increased discharge of PZP from the liver was found to be associated with activation of brown adipocytes in mouse models^25^.

Other significant proteins are well-known to be associated with IR. Similar to our prediabetic cohort, another longitudinal study (*n* = 90) reported Lipoprotein-a (LPA) to be reduced in the period preceding new-onset diabetes and to be inversely associated with the HOMA index^26^. Adiponectin (AdipoQ) is known to increase insulin’s ability to stimulate glucose uptake by increasing total GLUT4 expression (Fig. 5a). Apolipoprotein A-I proteins are constituents of high-density lipoprotein (HDL) that are found to be associated with obesity and metabolic syndrome in humans^27^. Overexpression of ApoD in transgenic mice was shown to increase insulin sensitivity by reduction in fat accumulation and enhanced energy expenditure^27,28^, similarly we also see higher ApoD intensity in IS individuals (Fig. 5a). Multiple studies involving animal models and human serum measurement have reported that the decreased level of CNDP1 is associated with insulin resistance^29,30^.

Beyond the healthy baseline, we next investigated the proteome change after respiratory viral infection (RVI). We identify 13 proteins (Fig. 5c) that change during the infection using DIAlignR—compared to eight proteins using the unaligned data (Supplementary Tables 10 and 11). As expected, most of them are involved in the viral response pathways. Three proteins SERPINA5, C9, and CPB2 are involved in the *complement and coagulation cascades* (KEGG id = hsa04610)^31^. The six proteins LRG1, SAA1, LBP, CPN2, C9, and CPB2 are represented in the *innate immune response pathway* (id = R-HSA-168249). During infection, the level of SAA1 increases, which activates and recruits neutrophils to lungs^32^. In this phase, SAA1 also displaces plasma ApoA-I proteins to become a major apolipoprotein of HDL^33^. Consistent with this argument, we do observe lower concentration of APOC3 and APOA4 in Fig. 5c. GPLD1 is a known physical interacting partner of APOA4, which is also found to be downregulated in the early days of infection^34^. CNDP1 (Carnosine80 Dipeptidase 1) encodes a metalloprotease protein which degrades carnosine that is known to modulate neutrophils with respect to respiratory burst for pathogen killing^35^. Lower CNDP1 levels have also been observed in serum of children infected with respiratory syncytial virus^36^. Both LUM and IL1RAP encoded proteins play an important role in the regulation of innate immunity^37^. However, to our knowledge, their circulating level in plasma has not yet been associated with viral infection.

Next, we wanted to see if the improved quantitation can also help in identifying proteome dynamics during RVI. We detect two temporal clusters that are different from the unaligned data (Figs. 5d and S28c). Genes found in cluster 1 are associated to the same pathway identified from pre-aligned data, however, the temporal pattern of these proteins slightly differ. Cluster 2 from the unaligned data could not be associated with any pathway. Interestingly, this cluster from the aligned matrix has eight genes that participate in VDJ recombination^38^. Possibly, these proteins belong to antibodies secreted from plasma cells, however the reason for the observed abundance-pattern during the infection is unclear and requires further investigation. The core genes for each cluster are mentioned in Supplementary Tables 12 and13.

In conclusion, the results derived from SWATH-MS data and analyzed with our novel signal alignment pipeline not only are consistent with the literature on insulin resistance and viral infection response, but additionally, are able to uncover new potential biomarkers and correlating proteins for IR and RVI response, respectively.

## Discussion

Tools such as TRIC^8^ often falter with multisite data, and others like Skyline demand manual alignment^39^. Addressing a notable gap in the automated analysis of large-scale DIA runs, the DIAlignR workflow (>2.3) conducts alignment across hundreds of proteomic LC-MS/MS runs. By combining the peak-picking (OpenMS) and peak- scoring (OpenSWATH & PyProphet) with signal alignment by DIAlignR, we demonstrate the improvement in the quantitative accuracy of the peptide-intensity tables on four complex datasets, two of which were acquired under highly heterogeneous conditions. Besides displaying greater accuracy compared to unaligned tables on manual annotated-peaks, we also show improved quantitative reproducibility with DIAlignR workflow compared to the TRIC software on a challenging multisite dataset.

Commonly used strategies for aligning multiple LC-MS/MS runs include Star or MST based approaches. In addition to these methods, we have also implemented a reference-free Progressive alignment in the workflow. Using signal alignment, we are able to improve the peak-selection process, reducing the error-rate to almost three-fold and recapturing missing/removed peaks, hence improving the overall quality of the data-matrix. On a homogenous dataset, the peaks generated from RT mapping can complete the peptide-intensity table close to 100% at the expense of mild increment in the quantitation error-rate from commonly used 1 to 5%. Nonetheless, inclusion of such peaks may not always be desired and additional research is warranted on how to handle peaks that are close to the noise threshold.

The implementation for Star and MST alignment in DIAlignR does not introduce bias toward a specific run as the reference run is different for each peptide. However, these methods are not able to fully incorporate information from all runs while selecting a reference, which is truly achieved by the Progressive alignment. The latter generates a template chromatogram with reproducible signal around a peptide-peak which is useful not only for manual annotations in standard tools such as Skyline, but also for manipulating chromatogram libraries^40^. In some cases, the differential signal can be easily visualized in the context of nearby peaks. Since RT shift was mild in the homogenous validation data, all three multirun strategies gave similar error-rates with hybrid pairwise-alignment.

Our evaluation on the multi-species dataset revealed that DIAlignR effectively controls the quantitative error rate and improves precision by reducing the coefficient of variation (CV) in protein intensities for *E. coli*, Human, and Yeast. Additionally, DIAlignR demonstrated its ability to accurately align peaks and improve the precision of expected species ratios, resulting in reduced variances and tighter variances at higher FDR thresholds. These findings provide a comprehensive understanding of DIAlignR’s impact on quantitative measurements and its capability to handle peak addition and removal effectively. Specifically in cases where the interest is in detecting changes in the small set of proteins that are important in discerning different sample comparisons.

During the multisite analysis, it became apparent that the differences amongst multirun methods stem from the global fits employed in the hybrid alignment. Although all three methods report lower CV compared to the unaligned table, we find that in the case of multi-column heterogeneity, MST performs better than Star and Progressive methods. Based on these observations, we have summarized the features of each multirun method in Supplementary Table 5. Apart from evaluating multirun methods, this dataset highlights the limitations of currently used tools such as TRIC in analyzing such heterogeneous data, and why many large-scale proteomics studies have avoided cross-run RT alignment. We also observe a large increment in CV (about 66%) from site-specific to cross-site analysis (Table 1), highlighting the challenges in multisite experiments which to a little extent are ameliorated by DIAlignR workflow. However, other issues such as normalization coefficient, peak-saturation, peak-shouldering, and irregular peak-boundary also affect quantitation and need to be addressed for automated analysis of label-free experiments.

A 2009 shotgun proteomics study^41^ found a large disparity between labs in protein identification from various samples (NCI-20, Sigma UPS 1, Yeast lysate). However, the differences were reduced with extensive separation before LC-MS/MS^42^. In comparison, our workflow consistently produces accurate protein quantification. Another MRM study measured 22 peptides across 15 sites in 10 replicates using heavy labeled internal standards, and found an average 9% site-specific CV^43^. On the other hand, our workflow dramatically increases the number of precursor ions and eliminates the need for internal standards. When applied to 229 runs of a human cell line sample, it fully quantifies 8509 precursors out of 52k precursors in every sample at 1% quantitation FDR, yielding a median site-specific CV of 9.3% (Supplementary Table 3). Across all sites, the cross-site CV was 19.8%, which meets clinical standards^44^. While the accuracy may be slightly lower when calculated for all identified analytes, it is a notable achievement for large-scale label-free studies for producing reliable results without the expensive calibration reagents across multiple instruments.

Our approach scales well to large-scale datasets (Supplementary Note 9 and Supplementary Table 14), allowing us to easily align 900+ plasma runs. By reanalyzing the prediabetic cohort data using our new workflow, we are able to detect many proteins associated with insulin resistance that were not reported in the original publication. Prior studies using large-scale DIA have not employed cross-run RT alignment to improve quantitativeness. Although demonstrated with two models: LDA and XGBoost from PyProphet, the open-source algorithm of DIAlignR would be a valuable addition to other scoring methods, as most of the tools do not use neighborhood context while scoring each peak. Alignment improves peak-selection, leading to enhanced biological insights in complex diseases. Besides correcting for chromatographic artifacts, we have also factored-in the mass-spectrometer sensitivity (run order) and batch effects in our differential analysis model. These strategies help in reducing the overhead of spiked-in standards and technical replicates, which are commonly used in other large-scale studies.

Data acquisition across different instruments, chromatographic setups or even sites has been a long-standing challenge in the proteomics community. The DIA scheme- along with DIAlignR workflow- is capable of identifying and quantifying peptides across such heterogeneous conditions, enabling multi-instrument and multisite label-free proteomics studies in the future. Besides clinical proteomics, this is significant for the developing field of single-cell proteomics where the analysis of hundreds-to-thousands of cells is essential for quantifying biological heterogeneity^45^. DIAlignR is available on Bioconductor, and can be integrated into a multitude of proteomics software. Our approach is reagent-free, generalizable and easily transferable to existing SWATH or DIA datasets. DIA records fragments of all ionized molecules, however, this unique feature has not been exploited for multisite analysis; with the DIAlignR workflow we demonstrate this capability. Thus, we hope, it will encourage the community to parallelize DIA measurements for larger data-acquisitions.

## Methods

### Spectra files

The instrument generated files were converted to mzML using MSConvert (docker: chambm/pwiz-skyline-i-agree-to-the-vendor-licenses:c30f8e5beb5f) without peak-picking. The plasma files already had linear compression for *m*/*z* and positive integer compression for intensity. The *S. Pyogenes* cell lysate files are available at PASS01508, the multi-species data are from PXD002952, the multisite data are from PXD004886, and clinical plasma files are downloaded from the iPOP portal.

### DIA Library

For the *S. Pyogenes* cell lysate analysis, the original library was downloaded from PASS00788. For the multi-species dataset, the library was downloaded from PXD004886, whereas for the multisite data, the pan-human library was considered in which assays for 11 iRTs and 30 AQUA peptides were added. The library for clinical plasma dataset is based on the UK twin plasma study. In the library, ids from 11 iRTs and PBMC samples were added, and peptide sequences were corrected based on Uniprot sequences available in December 2021. The sequences not matching the uniprot database were manually removed.

All libraries were modified to have the same retention time for multiple charge states of peptides. Peptides with NormalizedRetentionTime different >4 for different charge states were removed. For other peptides, the NormalizedRetentionTime was averaged across different charge states. The *S. Pyogenes* library is available at PASS01508. Libraries used for the multisite analysis and plasma data analysis are uploaded on Zenodo repo 6677715.

### Peak-picking using OpenMS

The spectra files were parsed with corresponding library and OpenSWATH (docker: openms/executables:84191d62898d). If not explicitly specified, the following parameters were used: RT extraction window = 600 s, extra RT window = 50 s, min_upper_edge_dist = 1, MS2 extraction window = 75 ppm, MS1 extraction window = 35 ppm, DIA extraction window = 75 ppm, ppm quadratic regression for mass correction, background subtraction with vertical_division_min for peak-intensity, mutual information and MS1 scoring were added. OpenSWATH produces feature files (.osw) and XICs (.chrom.mzML). The produced mzML chromatograms were then converted to chrom.sqMass files with OpenSwathMzMLFileCacher and lossy-compression set to false. The specific parameters for each experiment are in the Supplementary Notes 5–8. The script is available at the DIAlignR wiki.

### Peak-scoring using PyProphet

Features from bacterial cell lysates are merged into a merged.osw, whereas for the multisite and plasma data, the features are processed without creating a merged file in PyProphet (docker: pyprophet/pyprophet:2.1.10). Features are scored using XGBoost classifier with Level=ms1ms2, initial FDR = 0.01, and integration FDR = 0.05. FDR for peak groups and peptides are termed as *mscore* and *q* value, respectively in the manuscript. The specific parameters for each experiment are in the Supplementary Text.

### Data-matrix using DIAlignR

The *S. Pyogenes* data were aligned with alignTargetedRuns, mstAlignRuns, and progAlignRuns for Star, MST and Progressive alignment respectively. For the larger datasets, multisite and plasma sample, peptides were divided into 10 fractions to distribute the computation across multiple cpus. The source-code with specific parameters for each experiment is available at the DIAlignR wiki.

To perform alignment, first default parameters were obtained using *paramsDIAlignR()*. Parameters *transitionIntensity* and *hardConstrain* were set to True, *maxFdrQuery*, *alignedFDR1*, and *alignedFDR2* were set to 0.05. Minimum spanning tree based alignment was performed for multirun alignment. The final data matrix was filtered with *mscore* ≤ 0.025 and *q* value ≤ 0.025 with signal integration within run enabled.

### Data-matrix using TRIC

In TRIC (docker: shubham1637/msproteomicstools:0.11.0), the feature_alignment.py function was used with readmethod = cminimal, realign_method = lowess_cython, mst:Stdev_multiplier = 4.0, and mst:useRTCorrection set to True.

### *S. Pyogenes* data analysis

The intensities were median-normalized and log2 transformed, and technical replicates labeled as R01 were discarded from downstream analysis. The *S. Pyogenes* protein sequences (UP000000750_301447.fasta) were downloaded in October 2021. Peptides that mapped to the genome were kept. Fragment-ions quantified in 60% of runs were retained. Top-3 fragment ions per peptide and top-3 peptides per protein were selected for differential analysis. Singleton proteins, with one peptide, were discarded from the analysis resulting in 1001 proteins. For each protein, the following model was used for ANOVA:

$${{{{{{\rm{stats}}}}}}}::{{{{{{\rm{aov}}}}}}}({{{{{{\rm{intensity}}}}}}} \sim {{{{{{\rm{bioRep}}}}}}}+{{{{{{\rm{peptide}}}}}}}+{{{{{{\rm{condition}}}}}}},{{{{{{\rm{df}}}}}}})$$

where df is a table that has log2 normalized intensity of each peptide and biorep ID of each run; condition refers to 0% or 10% plasma added during bacterial growth. The *p* values derived from ANOVA were adjusted by the Benjamini-Hochberg correction for multiple comparisons. Proteins with adjusted *p* values ≤ 0.05 and |effect size| > 1 were considered to have differential abundance. Genes were visualized in PATRIC^46^ version3.6.12 for *Streptococcus pyogenes* M1 476 strain (accession number AP012491). The protein protein interaction networks were fetched from the STRING db^47^.

### Quantitation error-rate

This value indicates the ratio of false-peaks to true-peaks from all runs used for peptide quantification in the data-matrix. In the absence of manual annotations, we use *q* value from PyProphet results to control for this error-rate^8,14^. Besides *q* value, PyProphet has an additional *mscore* filter to keep only high-quality peaks. Unless explicitly specified, both filters are assigned identical values. When compared to manual annotations, a peak was considered true if it overlaps with the annotated one. Peaks picked below a *q* value threshold but missing in manual annotations are called incorrect. Peaks resulting from signal integrations (with no *q* value), but missing in manual annotations are excluded from error-rate calculation. The error-rate is calculated as:

$${{{{{{{\rm{Quantitation}}}}}}}\,{{{{{{\rm{error}}}}}}}\,{{{{{{\rm{rate}}}}}}}}_{{{q}{{{{{{\rm{value}}}}}}}}}=\frac{{{{{{{{\rm{Correct}}}}}}}\,{{{{{{\rm{peaks}}}}}}}}_{{{q}{{{{{{\rm{value}}}}}}}}}}{{{{{{{{\rm{Total}}}}}}}\,{{{{{{\rm{peaks}}}}}}}}_{{{q}{{{{{{\rm{value}}}}}}}}}}$$

### Multisite data analysis

The intensities were normalized with the table used in the original publication. For peptides, top six fragment-ions are used, selected without alignment. Protein quantification is done using top 3 peptides and their top 5 fragment-ions. Coefficient of Variation (CV) was calculated as standard deviation divided by mean of intensity for an analyte.

### Clinical plasma data analysis

Runs having less than 4950 transitions (= ${\mu }_{n}$ − 1.96*${{{{{{{\rm{sd}}}}}}}}_{n}$) were removed. Three other runs were removed due to low total ion signals. In the remaining 925 runs, transitions quantified in at least 40% of runs were kept. Peptide intensities were median normalized. Peptide abundance is inferred by summing top 5 most intense transitions. Protein quantitation is done by summing the top three peptides. The transitions, peptides used for quantification, runs selected for analysis and their normalization coefficients are provided in the Zenodo repo. Intensities were log2 transformed. The final data-matrix had 227 proteins across 925 runs with 85% completeness.

#### Differential proteomics

Only healthy samples from known IR and IS participants (*n* = 416) were used to find associated proteins. Following model was used for each protein:

$${{{nlme}}} 	::{{{lme}}}({{{intensity}}} \sim {{{IRIS}}}+{{{peptideID}}},\\ {{{random}}} 	={{{list}}}( \sim 1{{{{{\rm{|}}}}}}{{{Batch}}}, \sim 1{{{{{\rm{|}}}}}}{{{AcqOrder}}}, \sim 1{{{{{\rm{|}}}}}}{{{ID}}}),{{{method}}}={{{ML}}})$$

where IRIS is the status of each participant (ID), Batch is the factor variable of the sample, AcqOrder is the acquisition order of the sample in that batch. The *p* value was obtained by comparing the above model with the NULL model using anova(). The effect size is generated by fitting the above model with method = REML. A protein is called significant if its effect size >log2(1.25) and BH-corrected *p* value ≤ 0.05. These proteins are mentioned in Fig. 5, and also used to determine any bias by DIAlignR in Fig. S29. To visualize the differential protein abundance (Fig. S28a), we removed batch and acquisition order, and participant specific effects from each sample. Following model was used to obtain their coefficients for each peptide:

$${{{nlme}}} 	::{{{lme}}}({{{intensity}}} \sim 0+{{{Batch}}}+{{{Batch}}}:{{{AcqOrder}}},\\ {{{random}}} 	={{{list}}}( \sim 1{{{{{\rm{|}}}}}}{{{IRIS}}}, \sim 1{{{{{\rm{|}}}}}}{{{ID}}}),{{{method}}}={{{REML}}})$$

#### Longitudinal analysis

There are 411 samples to determine proteome change during RVI. To investigate proteins that changed, following model was used:

$${{{nlme}}} 	::{{{lme}}}({{{intensity}}} \sim {{{event}}}+{{{peptideID}}},\\ {{{random}}} 	={{{list}}}( \sim 1{{{{{\rm{|}}}}}}{{{Batch}}}, \sim 1{{{{{\rm{|}}}}}}{{{AcqOrder}}}, \sim 1{{{{{\rm{|}}}}}}{{{ID}}}),{{{method}}}={{{ML}}})$$

where event is a factor variable with five levels as described above. The *p* value for each protein was obtained by comparing the above model with the NULL model using anova(). A protein is called significant if its BH-corrected *p* value ≤ 0.05.

The fuzzy c-means clustering was performed to recognize the longitudinal patterns^48^. We used the elbow method to identify the optimal number of clusters(= 4) in our data set. The data was standardized to *z*-scores for each peptide and subjected to c-means clustering over the course of RVI. We used a minimum *acore* as 0.6 to get the core proteins of each cluster. To identify the function of these proteins, we used the IMPaLA^49^ tool pathway over-representation analysis with *q* value = 1.0.

### Statistics and reproducibility

To identify associated proteins, we have performed linear mixed-effect models. In addition, all the data used are public datasets and all the codes used are publicly available at Github to guarantee the reproducibility of all the experiments.

### Reporting summary

Further information on research design is available in the Nature Portfolio Reporting Summary linked to this article.

### Supplementary information

Supplementary Text

Description of Additional Supplementary Files

Supplemental Data

Reporting Summary

## Data Availability

For manual annotation and analysis of bacterial growth, we have used previously published data from PASS01508. For multi-species analysis, we use data previously published and available from PXD002952. The chromatograms, features and other results of this paper are added in the *manualAnnotation* and *differentialAnalysis* directories. Multisite data and clinical plasma dataset were fetched from PXD004886 and the iPOP portal, respectively. The libraries used in their analysis and results are uploaded to Zenodo (zenodo.org/record/6677715) in restricted mode and can be fetched with this link. The Supplementary figures and tables are in Supplementary Text. The numerical data behind the graphs in the figures is available in the Supplementary Data file.

## Code availability

DIAlignR is open-source and is freely available at https://github.com/shubham1637/DIAlignR under a GPL-3 license.
