# Supplementary material for: Achieving quantitative reproducibility in label-free multisite DIA experiments through multirun alignment
Source: Commun Biol. 2023 Oct 30;6:1101. doi: 10.1038/s42003-023-05437-2 (PMC10616189; doi:10.1038/s42003-023-05437-2)
Supplement: Supplementary file 1 — Supplementary Text [file 42003_2023_5437_MOESM1_ESM.pdf]

Supplementary Text

Achieving Quantitative Reproducibility in Label-Free Multisite DIA Experiments Through Multirun Alignment

Authors:
Shubham Gupta[1,2], Justin C. Sing[1,2], Hannes L. Röst*[1,2,3]
1. Terrence Donnelly Centre for Cellular & Biomolecular Research, University of Toronto, Toronto, Canada.
2. Department of Molecular Genetics, University of Toronto, Toronto, Canada
3. Department of Computer Science, University of Toronto, Toronto, Canada

* Corresponding author: hannes.rost@utoronto.ca

Supplementary Notes:

Supplementary Note 1: Pairwise Retention Time Alignment
Supplementary Note 2: Overview of Multirun Alignment using DIAlignR
- 1. Tree construction for MST and Progressive alignment
- 2. Star Alignment
- 3. MST traversal
- 4. Hierarchical tree traversal
- 5. Pairwise alignment
- 6. Peak selection and signal integration

Supplementary Note 3: Creating Master Runs for Progressive Alignment
- 1. Chromatogram merging
- 2. Feature picking and scoring

Supplementary Note 4: Parameter optimization for MST and progressive alignment
- 1. Distance Metric for MST
- 2. Progressive alignment parameters

Supplementary Note 5: Gold Standard Manual Annotation Data
- 1. MSConvert + OpenSWATH + PyProphet
- 2. DIAlignR
- 3. Precision-Recall
- 4. Retention time (RT) error
- 5. qvalue control with signal integration across runs

Supplementary Note 6: Multisite 229 HEK293 cell lysate runs
- 1. Data summary
- 2. MSConvert + OpenSWATH + PyProphet
- 3. Comparison to published results
- 4. DIAlignR
- 5. Across Sites alignment
- 6. Comparison of multi-run alignment methods

Supplementary Note 7: S. Pyogenes growth in plasma - differential proteomics analysis
1. MSConvert + OpenSWATH + PyProphet
2. DIAlignR
3. Differential expression
4. Chromatogram visualization
Supplementary Note 8: Prediabetic study - 949 human plasma runs
1. MSConvert + OpenSWATH + PyProphet
2. DIAlignR
3. Insulin resistant v/s insulin sensitive
4. Change in proteome during respiratory viral infection
5. Comparison with original paper
Supplementary Note 9: Software Versions
References

Supplementary Tables:

Name	Description	Page
1	Run acquisition information for *S. Pyogenes* data	16
2	Comparison of reanalysis of Multilab data to published results	20
3	CV of fully quantified precursors	24
4	Number of global alignments calculated	24
5	Comparison of multirun alignment methods	24
6	Results of differential proteomics analysis	27
7	Fold change and *p-value* before or after alignment	28
8	Fold change and *p-value* of proteins called significant before or after DIAlignR	32
9	Effect of FDR control on IR-IS associated proteins	34
10	*p-value* of significant proteins from RVI samples with DIAlignR	35
11	*p-value* of significant proteins from RVI samples without DIAlignR	35
12	Core genes in each cluster from aligned data	36
13	Core genes in each cluster from aligned data	36
14	Computational cost for TRIC and DIAlignR	37

Supplementary Figures:

Name	Description	Page
S1	Output of LC-MS/MS experiments: A quantitative data matrix	4
S2	Example trees for MST alignment and Progressive alignment	6
S3	Star alignment steps	6
S4	MST alignment steps	7
S5	Progressive alignment steps	8
S6	Detailed alignment strategies and comparison against manual annotation	10
S7	Merging of two chromatograms	11
S8	Minimum spanning tree by distance metrics	12
S9	FDR and peptides with incorrect peaks for different distance metrics	13
S10	Guide tree for *S. Pyogenes* data with NC distance	13
S11	Effect of distance metric, agglomeration strategy and strategies of alignment of runs	14
S12	Hierarchical clustering with heatmap obtained with NC distance	15
S13	Effect of including flanking chromatograms while creating merged chromatograms	15
S14	Effect of signal alignment on FDR v/s Recall	17
S15	RT error vs *mscore*	18
S16	RT error across annotated peaks	18
S17	ΔRT of the peptide peak and qvalue	19
S18	Recreation of published figures from Collins et al [6]	21
S19	Effect of DIAlignR on quantitation from multiside data	22
S20	Peak selection after signal alignment	23
S21	Comparison of Star, MST, and progressive alignment	25
S22	A quantification matrix from the SWATH-MS data before and after DIAlignR	27
S23	Volcano plot depicting proteins associated with bacterial growth in plasma	29
S24	Effect of alignment on peak-selection for *S. Pyogenes* analysis	29
S25	The connected protein networks from STRING	30
S26	The Genomic locus of *S. Pyogenes* depicting FAB proteins and virulence factors	30

S27	Fold change using all quantified peptides for three pathways	31
S28	Effect of alignment in the analysis of large-scale plasma proteome	33
S29	Effect of alignment on peak-selection for clinical plasma analysis	35

Abbreviations & Definitions:

CV Coefficient of Variation
DIA Data Independent Acquisition
FDR False Discovery Rate
IR Insulin Resistant
IS Insulin Sensitive
LDA Linear Discriminant Analysis
ML Machine Learning
MST Minimum Spanning Tree
RVI Respiratory Viral Infection
RT Retention Time
SSPG Steady state plasma glucose
SWATH-MS Sequential Window Acquisition of all THeoretical Mass Spectra
XIC Extracted Ion Chromatogram

dscore An aggregate discriminant score for discriminating Tagets from Decoys
mscore An FDR value (0, 1] for peaks scored by OpenSWATH+PyProphet
qvalue An FDR value (0, 1] for peptides scored by OpenSWATH+PyProphet

Supplementary Note 1: Pairwise Retention Time Alignment

One of the advantages of Data Independent Acquisition (DIA) is that it records signals from all the ionized molecules in an experiment. The data, thus, can be mined again with software as they evolve. We are presenting a state-of-the-art cross-run alignment tool, DIAlignR, which provides a more complete data-matrix compared to its predecessors.

A proteomic data-matrix produced in LC-MS/MS experiments has peptides in rows and samples in columns (Figure S1). An ideal data matrix would have quantification for each ionized peptide in all runs. In DIA, the multiplexed spectra result in noisy MS2 chromatograms, which makes it difficult to identify the correct peaks. Generally, DIA analysis software uses automated algorithms to identify regions of interest in a chromatogram [2]. They then use machine learning tools e.g. LDA, XGBoost, neural network ensemble etc to separate signal from noise and use statistical procedures to control the FDR [14,15]. Current software do not incorporate local context of peak in scoring, thus, prone to make mistakes when multiple good candidates are presented in a chromatogram. With FDR control, the error is controlled at the expense of a data-matrix with many missing values.

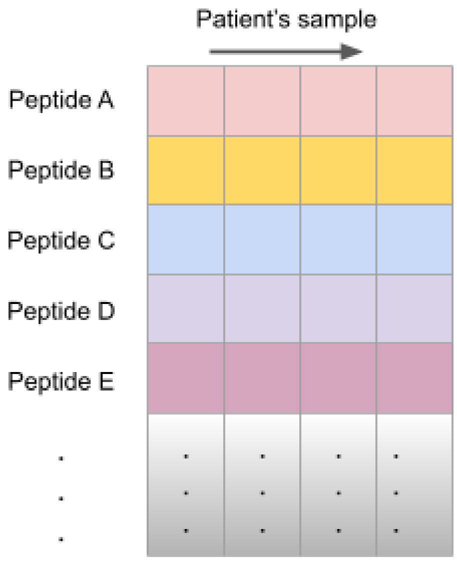

Figure S1. Output of LC-MS/MS experiments: A quantitative data matrix.

The problem of an incomplete data-matrix exacerbates in large-scale studies, especially when data is acquired at multiple sites. Since the retention time of peptides shifts unevenly across all peptides, machine learning tools struggle to factor peptide-specific variations into the scoring mechanism, resulting in a more sparse matrix. In consequence, these software promise accuracy at the expense of quantification events.

We argue that with signal alignment, we can add consistency in the peak-picking, hence, improving the accuracy further than what is promised by peak-scoring algorithms. Recently methods have been published that align retention time of peaks across DIA runs. LWBMatch and Group-DIA use MS1 chromatogram for coarse retention time alignment [10,11]. LWBMatch also uses MS2 features to

establish a bipartite matching, thus, avoiding monotonous fit imposed by MS1 alignment using dynamic programming. Nonetheless, MS1 signal is known to be more noisy than MS2 for SWATH-MS, that is why MS2 is preferred for quantitation as well [9]. With bipartite graphs, it is still reliant on OpenSWATH peak-picking and can not overcome the issues related to missing peaks.

Other tools have focused on MS2 peaks and use them to construct a linear or non-linear monotonous fit to map retention time of a peak from one run to another [5,12,13]. These methods work reasonably well and have been used in large-scale (100+ runs) experiments [5, 21]. However, they break-down when runs are acquired across multiple setups or different LC columns [1, 7].

Recently, we published a proof-of-concept *hybrid chromatogram alignment* method that uses raw MS2 chromatograms instead of MS2 peak-group features [1], termed as *signal alignment* here. Briefly, for each peptide a similarity matrix is calculated from MS2 chromatograms of two runs. The matrix is penalized using non-linear global fit, obtained from high-scoring common peaks, to constrain the alignment path. Then, with dynamic programming an alignment path is calculated that provides a retention time mapping for chromatograms. Since each peptide is aligned separately, this approach does not have monotonicity constraints and can align peptides that have switched elution order across runs [1]. In this paper, we are extending the pairwise *signal alignment* across multiple runs for peak selection, thus, improving the number of quantitation events at a certain FDR threshold.

Supplementary Note 2: Overview of Multirun Alignment using DIAlignR

Alignment of more than two runs involves guide-tree construction, seed run selection, pairwise alignment between two runs, peak selection etc depending on the strategy. We are explaining these steps below:

1. Tree construction for MST and Progressive alignment

Guide tree and hierarchical tree are needed for MST and progressive alignment, respectively. DIAlignR has an option to provide your own tree, e.g. based on acquisition order of run. However, an automated way for tree construction is preferred. The first step to build a tree is to calculate a pairwise distance matrix. We have implemented the following methods for a global distance matrix:

a) NC distance = $1 - 2\frac{N_{common}}{N_1 + N_2}$

   N_1,N_2 : Number of precursors having peaks with *mscore* below *analyteFDR* in run1 and run2.

   N_{common} : Number of precursors that are identified in both runs below *analyteFDR*.

b) RSE distance = Residual Standard Error (RSE) of non-linear RT fit.

c) R^2 distance = 1 - R^2 of linear fit.

DIAlignR uses an implementation of Kruskal's algorithm to get the MST from the distance matrix (Figure S2). For the hierarchical tree, UPGMA clustering is done on the distance matrix. MST is an undirected acyclic graph where each node represents an LC-MS/MS run. A hierarchical tree has LC-MS/MS runs as leaf nodes. Non-leaf nodes represent master runs which consist of a set of chromatograms and features for peptides. Each non-leaf node, including root, must have exactly two parent nodes. Root node is called *master1* by-default. DIAlignR has an option to provide your own tree, e.g., based on acquisition order of run, however, automated tree construction is preferred.

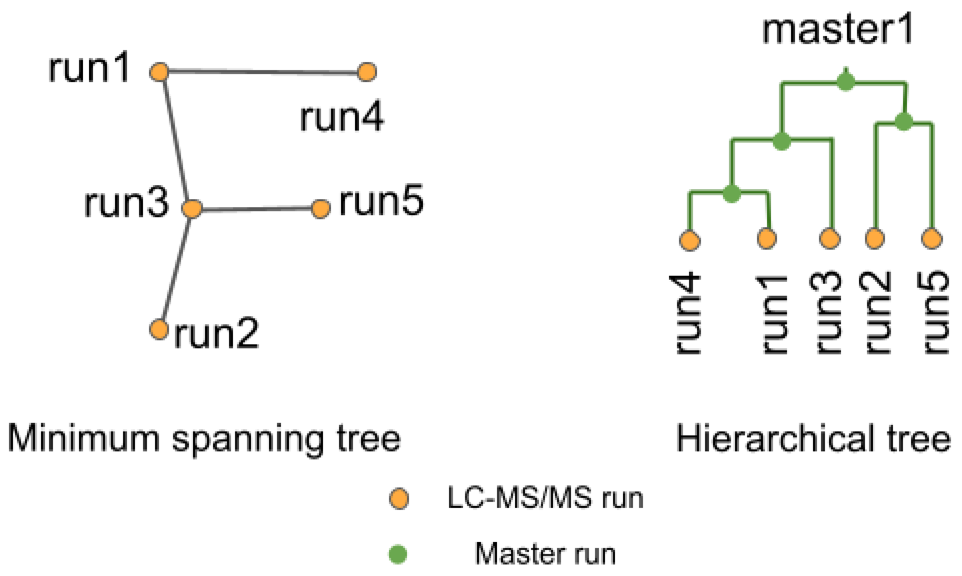

Figure S2: Example trees for MST alignment (left) and Progressive alignment (right).

2. Star Alignment

In Star alignment, first a reference run is selected for a peptide based on *qvalue* (Figure S3a) and the *alignment rank* is set to 1 for its peak with minimum *mscore*. The other runs are, successively, aligned to the reference run (Figure S3b) and alignment rank is set for the aligned peak (Figure S3c). The process is repeated for the rest of the peptides.

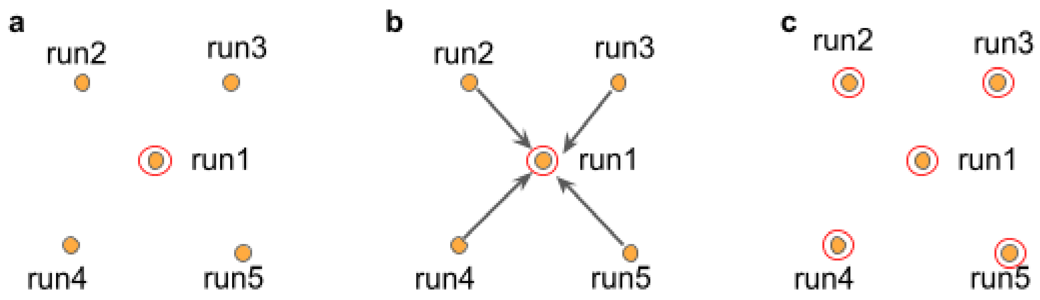

Figure S3: **Star alignment**. a) run1 is selected as a reference for a peptide and the *alignment rank* is set to 1 for its best-scoring peak. b) Other runs are aligned directly to the reference run1. c) Using retention time mapping from pairwise alignment, other runs also have peaks with *alignment rank* = 1.

3. MST traversal

Once a guide tree is built, a seed run is selected for each peptide like the Star method (Figure S4a). However, during the traversal only adjacent runs are aligned to the reference. Aligned peaks have their *alignment rank* set to 1 (Figure S4b). Subsequently, peaks with alignment rank become reference and adjacent nodes are aligned to them, till all nodes are visited. The steps are repeated for remaining peptides (Figure S4c,d).

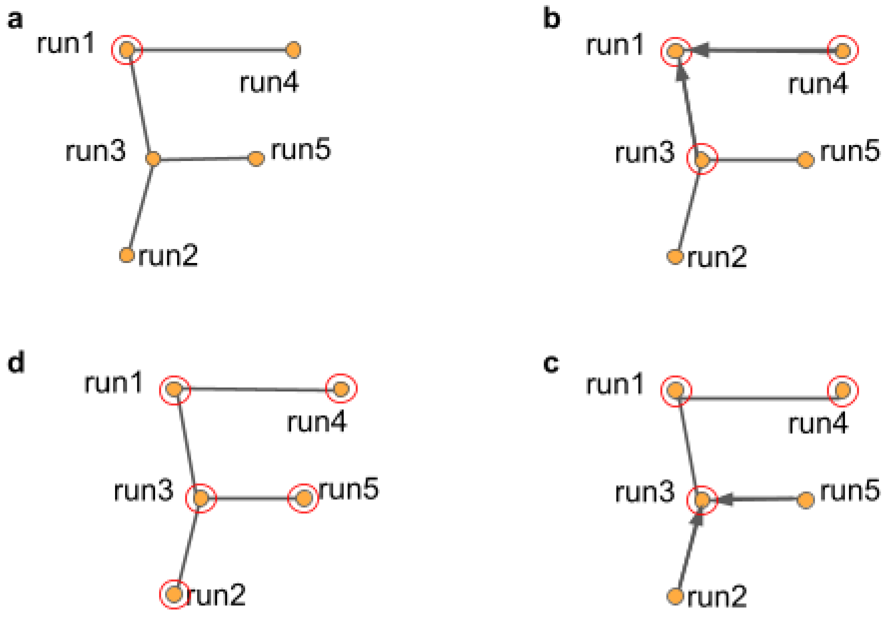

Figure S4: **MST alignment**. a) run1 is selected as a reference for a peptide and *alignment rank* is set to 1 for its best peak. b) Next, adjacent run3 and run4 are aligned to the run1. c) Alignment rank is set for run3 and run4, following that run2 and run5 are aligned to run3. d) In the end, all runs have peaks with *alignment rank* = 1, indicated by red circles.

4. Hierarchical tree traversal

In progressive alignment, as the hierarchical tree is traversed from the leaves to root, the master runs are generated and RT mapping are also saved (*_av.rds files). At the root there is **master1** run, in which for each peptide the peak with lowest *mscore* is set to have *alignment rank* = 1 (Figure S5c). The tree is, then, traversed from root to leaves. At this step, RT mapping is used to set *alignment rank* for leaf and non-leaf nodes.

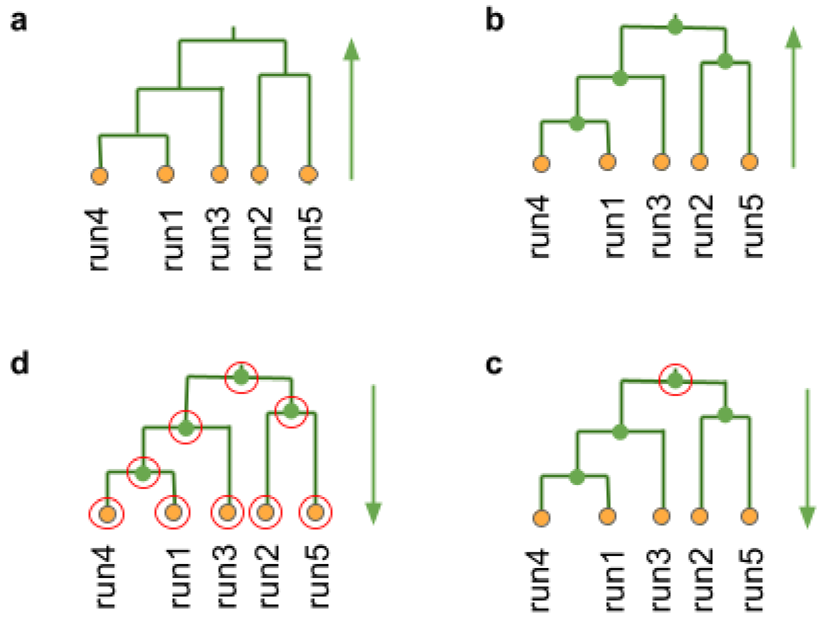

Figure S5: Progressive alignment. a) Tree is traversed from leaves to the root. b) Master runs are generated. c) At the root alignment rank is set for all peptides and d) peaks are then mapped to the LC-MS/MS runs.

5. Pairwise alignment

DIAlignR has three implementations [1] of pairwise alignment- 1) global, 2) local and 3) hybrid. Global alignment uses MS2 features below a certain *mscore* threshold to calculate either a linear or lowess fit. Local alignment uses extracted-ion-chromatograms (XICs) for each peptide, calculate a similarity matrix and find the alignment path using dynamic programming. Hybrid approach constrains the similarity matrix with global fit before performing dynamic programming, thus combining best of both local and global approaches.

6. Peak selection and signal integration

After pairwise alignment, peaks from the reference run are mapped to its counterpart (Figure S6a). If reference peak-boundary mapping overlaps a peak with *mscore* below a quality threshold, the peak's alignment rank is set. In case of multiple peaks with same score, the peak with the highest RT overlap is selected. If no good quality peak is found, the boundary is expanded by adaptiveRT. The peak with *mscore* below the quality threshold, if multiple then the lowest mscore, is selected and its alignment rank is set. If no peak is found within the expanded boundary, the signal within the aligned boundary is integrated; this is termed as **signal integration** (Figure 1b). The quantitation is done by OpenMS scripts to keep parity with the upstream analysis [ZZ]. Usually, a peak

picker fails when the signal is very close to noise, hence misses such low intensity features. Thus, a new feature is added with alignment rank = 1 and *mscore* = NA. To control the incorporation of signal-integrated peaks, I use *qvalue* control which is explained in Suppl Note 5.5.

Signal integration is carried out not only between runs, but also within a run. For peptides with multiple charge states, the precursor having lowest *mscore* peak is selected for the alignment. The peak boundaries from this precursor are mapped to other charge-states to pick peaks for related precursors; this is termed as **signal integration across charges**.

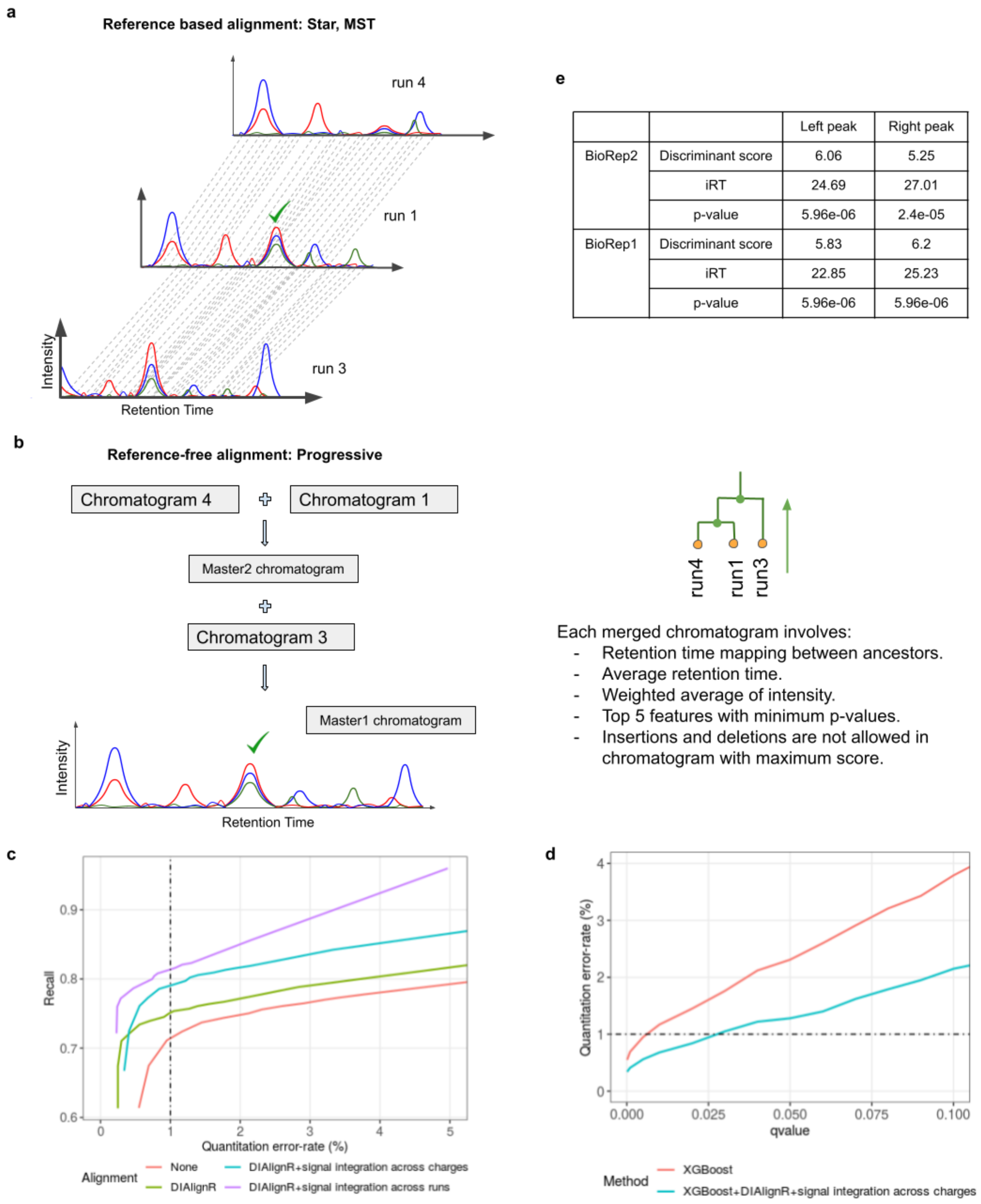

		Left peak	Right peak
BioRep2	Discriminant score	6.06	5.25
	iRT	24.69	27.01
	p-value	5.96e-06	2.4e-05
BioRep1	Discriminant score	5.83	6.2
	iRT	22.85	25.23
	p-value	5.96e-06	5.96e-06

Figure S6. a) Pictorial representation of Star and MST alignment. run4 and run3 are aligned to reference run1. RT mapping from hybrid alignment is depicted as gray lines. b) Merging the chromatograms from three runs provides a master chromatogram. c) The data-matrix completeness with the DIAlignR workflow. Different

ways to filter an alignment matrix are evaluated. For DIAlignR+signal Integration across runs, both *mscore* and *qvalue* (Suppl Note 5.5) control is used. The full-range figure is plotted in Figure S14a. d) DIAlignR workflow complements XGBoost in controlling error-rate. e) Scores of left and right peaks from Figure 1e.

Supplementary Note 3: Creating Master Runs for Progressive Alignment

In progresive alignment, two runs are merged to create a master run. The merging involves merging of chromatograms (sqMass or mzML files), merging of features and score calculation for them which happens in-memory.

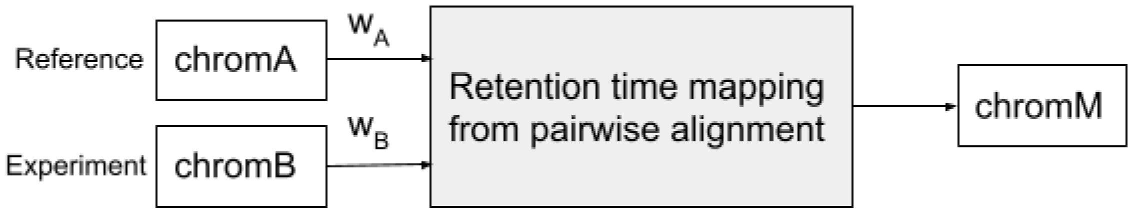

Figure S7: Merging of two chromatograms. Reference chromatogram chromA and Experiment chromatogram chromB are merged to output chromM. w_A and w_B are weights for intensity merging.

1. Chromatogram merging

Chromatograms have two components: time and intensity. With pairwise RT mapping the aligned region of chromatograms is weighted-averaged. The weight for each chromatogram is calculated as -log10 * $pvalue_{\text{experiment-wide}}$. Flanking regions from the ends of XICs are not mapped due to overlap alignment.

For the mapped regions of both reference, *chromA*, and experiment chromatograms, *chromB*, first retention time is linearly interpolated to fill gaps, and intensity is spline-interpolated (Figure S7). A merged chromatogram, *chromM*, is created with time being an average of time vectors, and intensity as weighted average of intensity vectors. In the merged chromatogram only those time points are kept for which there is no gap in the reference chromatogram. Next, flanking regions are added to the merged chromatogram. The intensity is appended unaltered, however, the retention time of the flanking region is modified based on the end-timepoints and sampling time of the merged chromatogram.

2. Feature picking and scoring

Features belonging to *chromA* and *chromB* are mapped in the *chromM*, and their *mscore* is assigned to new peaks as is. To avoid having duplicate/overlapping features, top five non-overlapping peaks with lowest *mscore* are selected. In case of the same *mscore*, the peak with higher intensity is

picked. The experiment-wide *qvalue* and *pvalue* of a peptide is set as the minimum of these from both runs.

Supplementary Note 4: Parameter optimization for MST and progressive alignment

We use manually annotated 437 peptides across 16 runs to get optimum parameters. The dataset is explained in the next section Suppl Note 5. The raw data and annotations are available in PeptideAtlas repository PASS01508.

1. Distance Metric for MST

The first parameter to optimize is the distance metric for guitde tree construction. We found that NC distance performs better than other measures as it constructs a tree where similar runs are clustered together (Figure S8-10). Overall the NC based MST provides lower error-rate compared to other measures. Out of 405 peptides compared, NC distance metric results in fewer peptides with incorrect peak-identification (Figure S9).

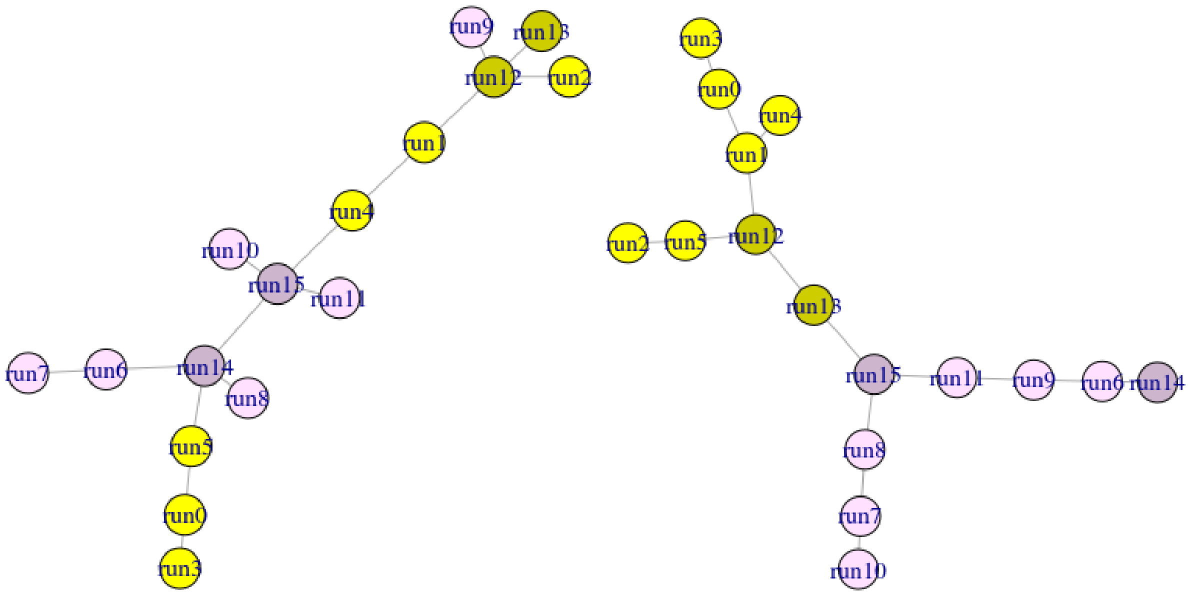

a) 1-R^2 distance b) NC distance

Figure S8: Minimum spanning tree by (1-R^2) distance metric (a) and NC distance metric (b). Yellow and purple colors represent 0% and 10% plasma in *S. Pyogenes* growth media. Light colored samples were acquired on Day 1, dark colored samples were acquired on Day 2.

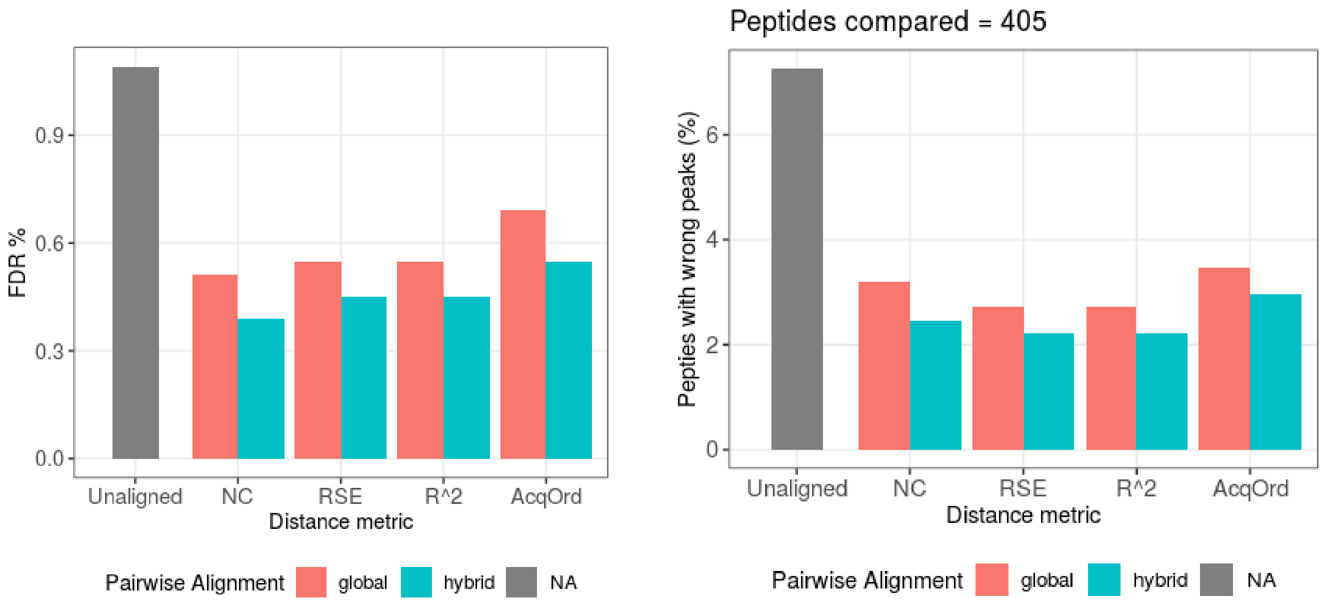

a) False Discovery Rate

b) Incorrect identified peptides

Figure S9. a) FDR for peaks identified at 1% *qvalue* by XGBoost when compared with manual annotation. b) Percentage of peptides, out of 405 annotated, having at least one incorrect peak.

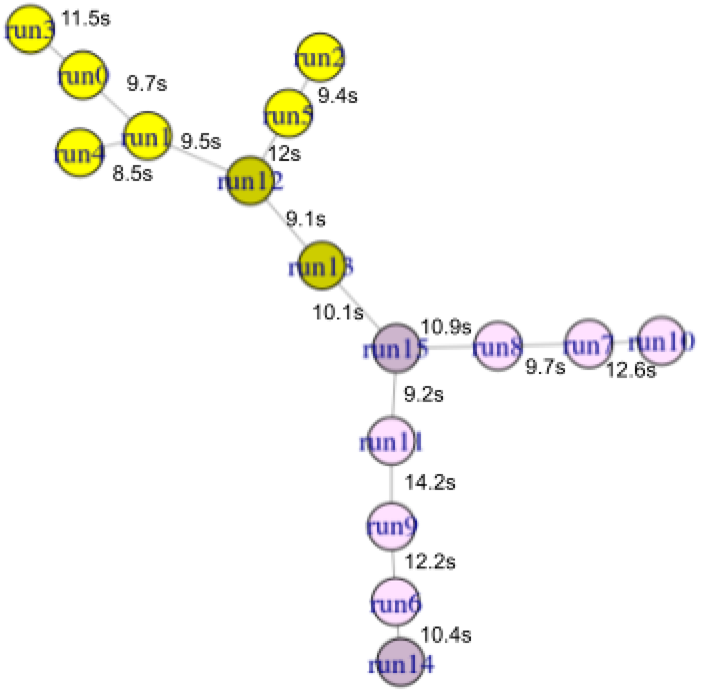

Figure S10. Guide tree for *S. Pyogenes* data with NC distance. RSE distance between runs is indicated for each edge.

2. Progressive alignment parameters

The aforementioned annotation data is used to get optimum parameters for progressive alignment. Following parameters are considered (optimal values are in boldface):

- Pairwise distance metric: R2, RSE, **NC distance**.
- Agglomeration strategy: Complete, Average, **Single linkage**.
- Align runs to master1 : direct align leaves to root, **Traverse tree and propagate alignment**.
- Aggregate p-values: Weighted average, **Minimum p-value**.
- Include flanking region in merged chromatograms: False, **True**.

After evaluating different pairwise distance metrics and agglomeration strategies for hierarchical clustering, we found that a tree constructed with NC distance measure and single linkage provides the lowest FDR (Figure S11). It is not surprising as a minimum-spanning-tree is equivalent to solving a single-linkage hierarchical clustering [16].

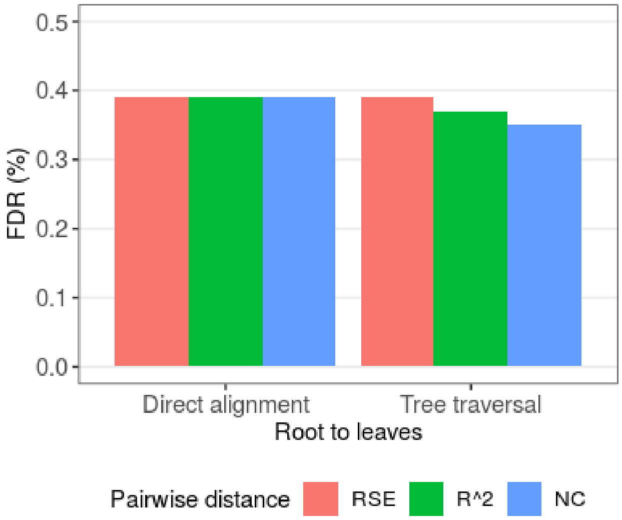

a) Selecting distance metric

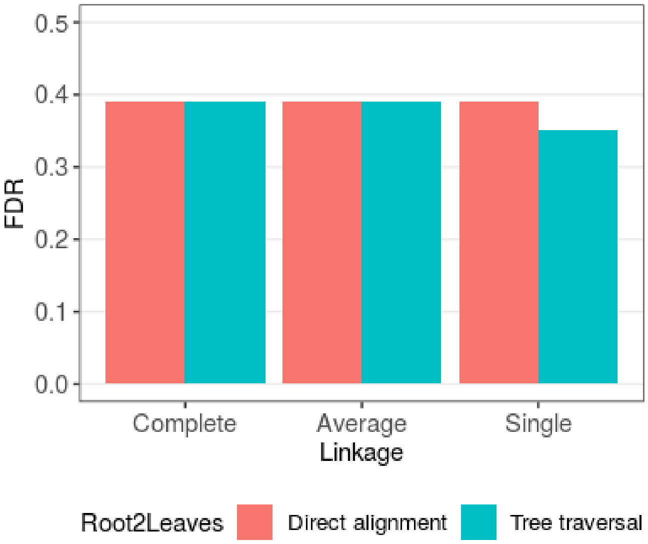

b) Aligning runs to master1

Figure S11. Effect of distance metric, agglomeration strategy and strategies of alignment of runs to master1. a) Three distance measures were compared for hybrid alignment. Two methods of setting *alignment rank* are evaluated after *alignment rank* is set in master1. b) Comparing three agglomeration strategies for hierarchical clustering.

Heatmap of the distance matrix with corresponding hierarchical clustering is presented in Figure S12. As with the MST clustering, similar runs are clustered together as can be seen on the colored strip on the left. run10 is an outlier as it does not cluster with any run; this is because the lowest number of common identifications at 1% *mscore*.

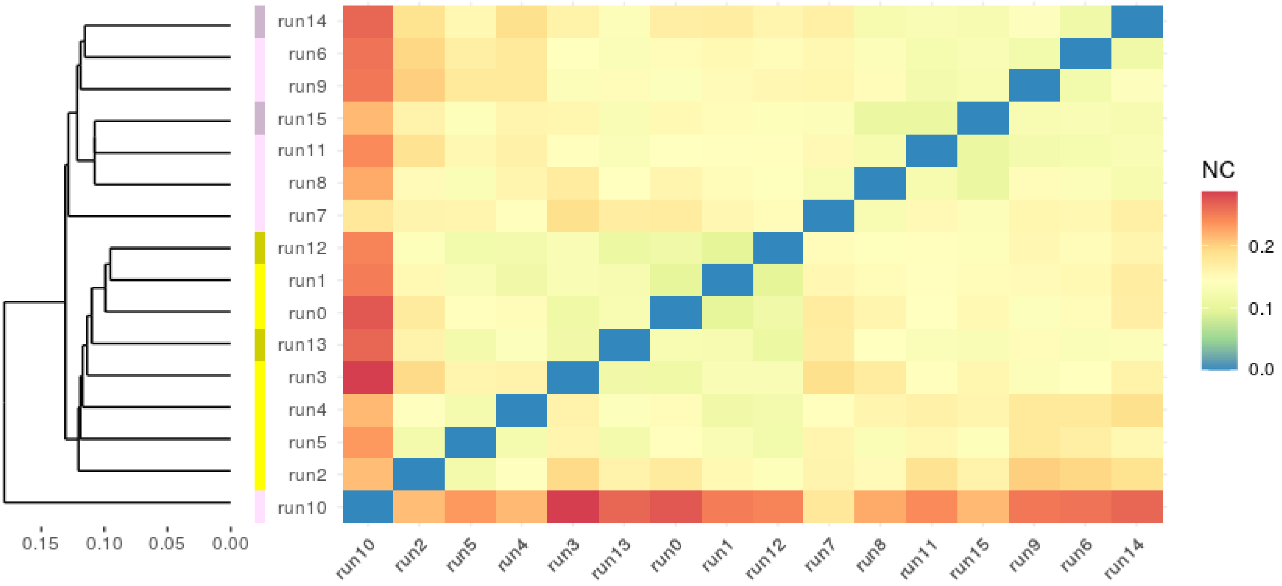

Figure S12. Hierarchical clustering with heatmap obtained with NC distance. Hierarchical tree is on the left with a scale indicating the agglomerative distance between clusters. The middle slice has color coding based on run ID. The heatmap on the right represents a pairwise distance matrix.

We next investigated *pvalue* aggregation method. *pvalue* are used for weighting intensities while merging chromatograms. The minimum of two p-values would be a conservative estimate, which also provides minimum FDR. We found that including the flanking chromatograms does help in alignment as there is more signal available for the subsequent alignment. Also, it would add more merged features which help in obtaining a better global fit (Figure S13).

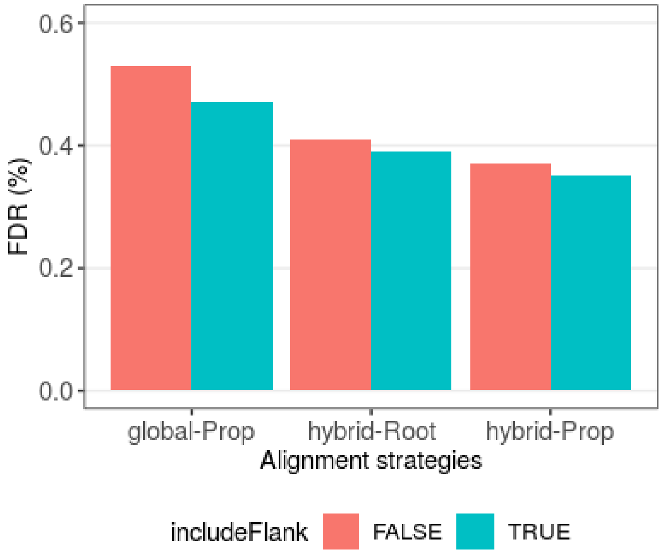

Figure S13. Effect of including flanking chromatograms while creating merged chromatograms. Compared its effect on both global and hybrid pairwise alignment. For hybrid alignment, both direct alignment to root and propagation *via* the tree is explored.

Supplementary Note 5: Gold Standard Manual Annotation Data

The details of library generation for the analysis of *S. Pyogenes* cell lysate data and data acquisition are available in Supplementary Note 6 of [4] and in [5]. Briefly, *S. Pyogenes* strain of M1 serotype was grown in 0% and 10% human plasma to investigate its growth in blood. There were a total of 16 DIA runs acquired with two biological replicates for each condition. In the previous iteration, 452 *S. Pyogenes* peptides were randomly selected and peaks were manually picked using Skyline (Supplementary Note 4 of [5]). Since, DIAlignR uses OpenSWATH extracted chromatograms, the peaks were re-annotated in these chromatograms using a Plotly script.

Supplementary Table 1: Run acquisition information for *S. Pyogenes* data

Run ID	Date	Plasma(%)	Biological Rep	Technical Rep
run0	2012-9-8	0	1	1
run1	2012-9-8	0	1	2
run2	2012-9-8	0	1	3
run3	2012-9-8	0	2	1
run4	2012-9-8	0	2	2
run5	2012-9-8	0	2	3
run6	2012-9-8	10	1	1
run7	2012-9-8	10	1	2
run8	2012-9-8	10	1	3
run9	2012-9-8	10	2	1
run10	2012-9-8	10	2	2
run11	2012-9-8	10	2	3
run12	2012-9-9	0	1	4
run13	2012-9-9	0	2	4
run14	2012-9-9	10	1	4
run15	2012-9-9	10	2	4

1. MSConvert + OpenSWATH + PyProphet

Wiff files were processed as described in [2]. Briefly, wiff files were converted to mzML with 64 -bit precision, numpress linear compression and vendor peakPicking using MSConvert version 3.0.21224. In OpenSWATH ms1 scoring, mutual information score and background_subtraction were set as true. In addition, a swath window file was used to specify isolation windows [4]. Lossy compression was set to False for converting chrom.mzML to chrom.sqMass files. For PyProphet score XGBoost classifier was used with ms1ms2 level, initial FDR set to 0.05 and iteration FDR set to 0.01. PyProphet combines OpenSWATH scores to an aggregate discriminant score which is then used to estimate *p-value* and *q-value* for each peak and peptide in run-specific, experiment-wide and global context [14]. *q-values* for peak groups and peptides are termed as *mscore* and *qvalue*, respectively.

2. DIAlignR

XGBoost scored features (osw) and OpenSWATH output chromatograms (sqMass) files are fed to DIAlignR to align all 16 runs. A range of 0.0001 to 1.0 *mscore* is used to investigate the effect of alignment in conjunction with XGBoost scores. Signal integrated peaks do not have corresponding *mscore* associated with them, hence, their inclusion is controlled with respective peptides' experiment-wide *qvalue* (explained in Note 5.5). With hybrid-progressive alignment, the number of incorrectly quantified peaks drops from 56 to 19 (Figure 1d), more than 60% reduction at 1% FDR.

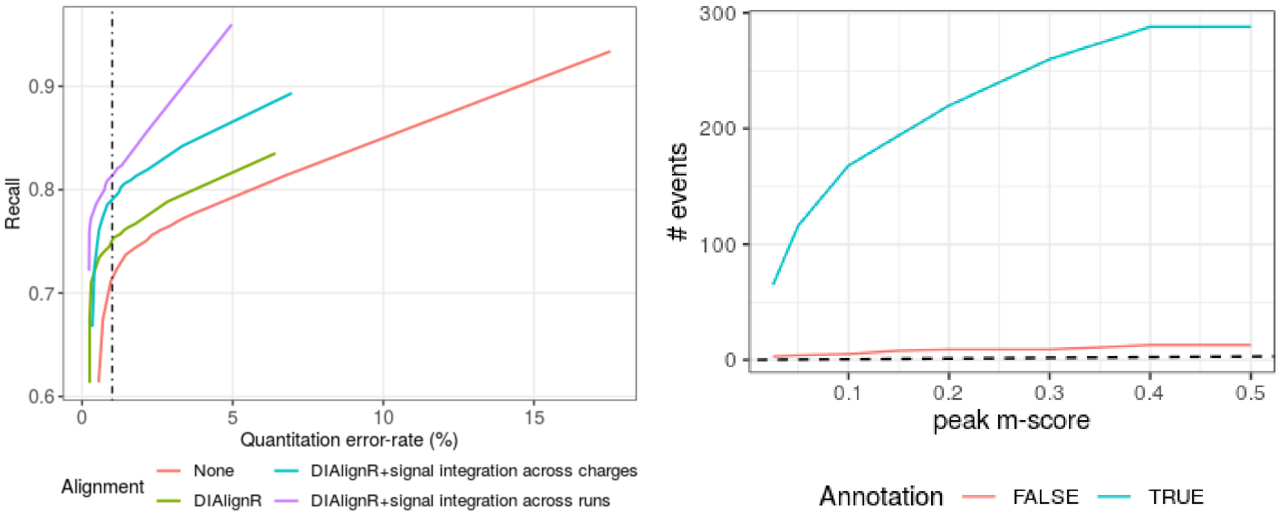

a) b)

Figure S14. a) Effect of signal alignment on FDR v/s Recall. The FDR-Recall plot compares an unaligned data matrix and various filtering of an aligned data matrix. The *None* alignment considers only *mscore* control. DIAlignR represents *mscore* control on aligned matrix, and signal integration includes peaks with missing *mscore*. FDR is calculated through manual annotations as described above. b) Newly created peaks that overlap with PyProphet m-score. Dashed-line indicates 1% FDR line.

3. Precision-Recall

As the *mscore* cutoff is increased from 0.0001 to 1.0, the number of correct peaks and total peaks increases. The Recall at a certain FDR level is depicted in figure above. The data matrix is obtained through pairwise hybrid alignment and star-based multirun alignment. Aligned matrix has better recall than unaligned at a given FDR. DIAlignR increases recall from 0.71 to 0.75 at 1% FDR. Being reliant on OpenSWATH peak-picking, it is unlikely to give 100% recall as peak-picker may fail to identify peaks in noisy chromatograms (Figure S14).

Signal integration across charge states fills some gap and increases recall from 0.83 to 0.9 at maximum FDR. For runs, where no peak is identified for a peptide, aligned peak-boundaries increase

the recall to 0.98. In remaining cases, aligned boundaries map out of the extracted-ion-chromatograms, hence, the signal cannot be quantified.

4. Retention time (RT) error

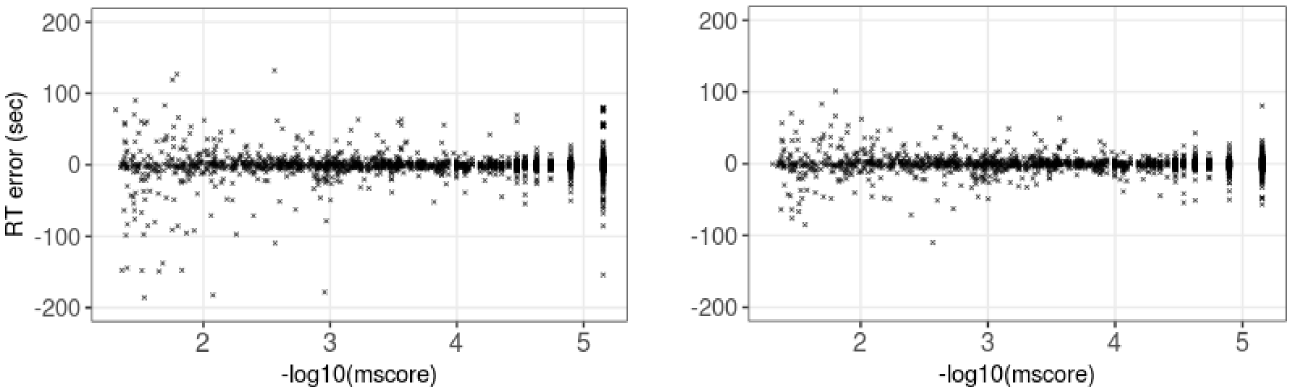

a) Without alignment b) With alignment

Figure S15. RT error vs *mscore*. a) XGBoost score only. b) XGBoost + DIAlignR.

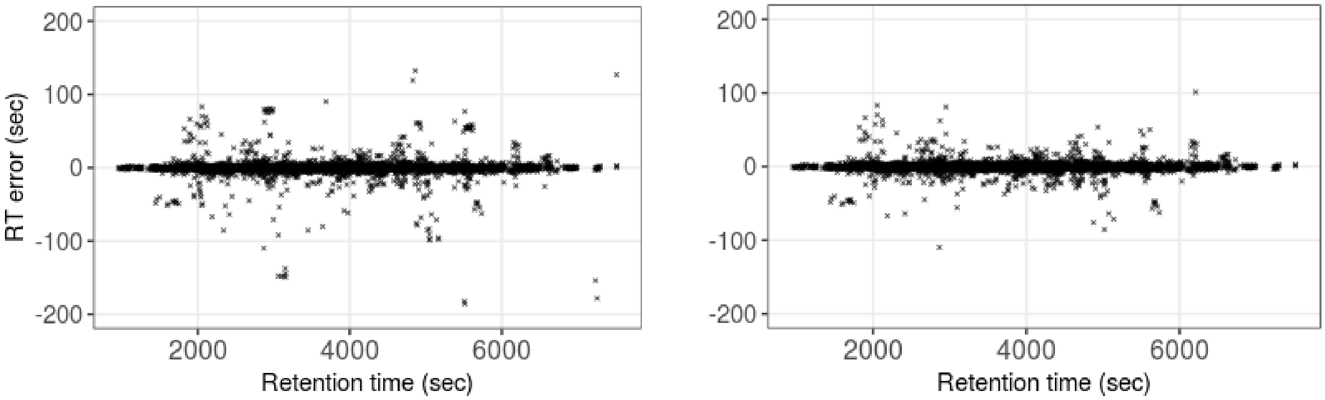

a) Without alignment b) With alignment

Figure S16. RT error across annotated peaks. X-axis shows retention times of manually annotated peaks. a) XGBoost score only. b) XGBoost + hybrid-star alignment.

Figure S15 depicts RT error of annotated peaks with and without alignment across the *qvalue* and *mscore* range of (0, 0.05]. Unsurprisingly, the spread is higher for peaks with high *mscore* due to lower confidence. In contrast, few high-confidence peaks also have high RT error. Nonetheless, signal alignment (Figure S15b) is able to correct the misaligned peaks. Figure S16 shows the retention time error across the RT range. Consistent with previous study [1], signal alignment reduces the RT error.

5. *qvalue* control with signal integration across runs

Peak creation (signal integration across runs) by mapping retention time from one run to another run is a contested topic [20] for the reason that there is no extensive scoring done while generating such features compared to peaks scored with PyProphet against decoys and hold a *p-value*. Hence, there is a need to control the error-rate arising from these new signal alignment based peaks.

We explored the peptide level *qvalue* to control the inclusion of such peaks in the quantitation matrix.

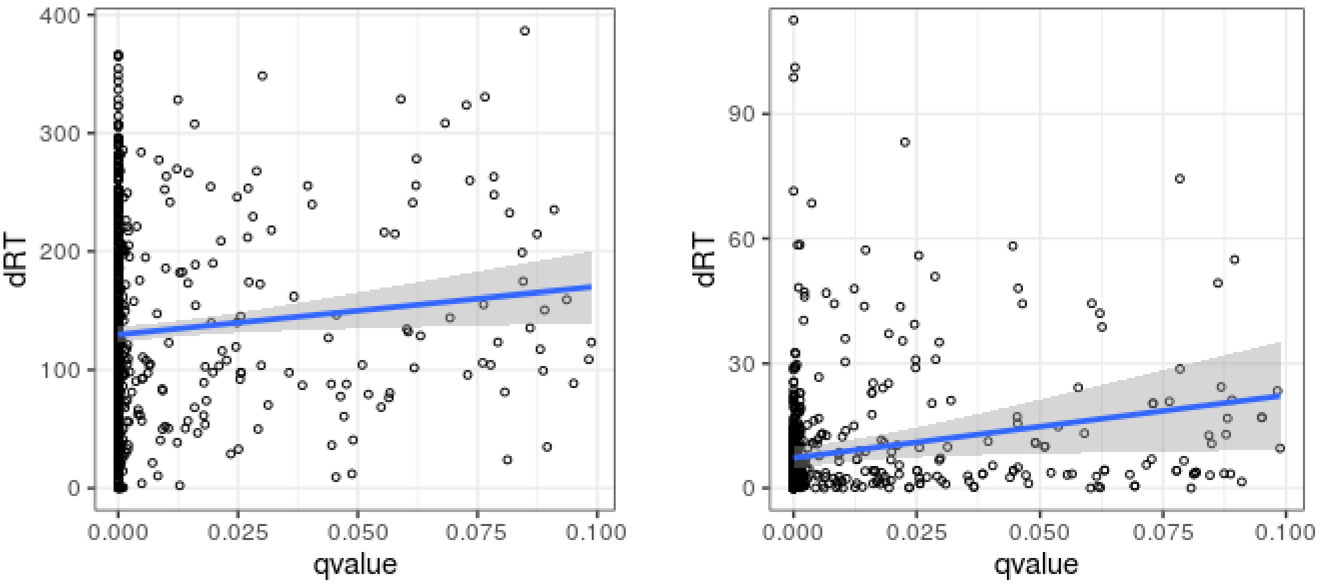

a) ΔRT with OpenSWATH peaks b) ΔRT with annotated peaks

Figure S17. ΔRT of the peptide peak and *qvalue*. a) ΔRT of aligned peaks with best scoring OpenSWATH peaks. PyProphet uses the best scoring peak to calculate *qvalue* for a peptide. b) ΔRT of aligned peaks with annotated peaks.

Generally, the aligned new peaks are farther from the best scoring peak picked by peak-picker, however, overall the peaks become more distant as *qvalue* increases (Figure S16a). Although the OpenSWATH peak is not correct, its *mscore* does reflect the retention time deviation (ΔiRT), which propagates to *qvalue*. On comparing manual annotation, we also observe that the new peaks are closer to ground truth for peptides with lower qvalues (Figure S17b).

Supplementary Note 6: Multisite 229 HEK293 cell lysate runs

This is a technical dataset which has 229 SWATH runs acquired using 11 LC-MS/MS setups. The sample had almost no biological variations.

1. Data summary

Digested peptides of HEK293 cell lysate, mixed with retention time calibration peptides from iRT-Kit (Biognosys) and 30 heavy labeled synthetic (AQUA) peptides were prepared for SWATH-MS acquisition. The AQUA peptides were divided into five groups (A-E) and each group had a concentration range to create the five different samples to be analyzed. Finally, samples were sent on dry ice to 11 sites. Each site acquired data for three days, seven samples per day on SCIEX TripleTOF 5600/5600+ systems, resulting in 21 samples per site. Total 229 data files were received for the

analysis. The detailed method for sample preparation, synthetic peptide concentration in each sample and data-acquisition is available in the original paper [6].

2. MSConvert + OpenSWATH + PyProphet

The analysis parameters were kept the same as done in the original paper [6]. Briefly, 229 wiff files were converted to mzML using MSConvert without peak-picking. For OpenSWATH following values are used: min_upper_edge_dist = 1, MS2 extraction window = 75 ppm, MS1 extraction window = 35 ppm, DIA extraction window = 75 ppm, RT extraction window = 900s, extra RT window = 100s, mutual information and MS1 scoring were added. Lossy compression was set to False for converting chrom.mzML to chrom.sqMass files. For PyProphet score, LDA classifier was used with ms1ms2 level and 0.4 value set for pi0_lambda.

3. Comparison to published results

The software OpenSWATH and PyProphet has evolved since the previous publication [6]. In addition to the modified library, there is a randomness involved in PyProphet scoring: a subset of features are selected for scaling up and ML classifier training. Nonetheless, the summary results are closely matching to published results (Supp Table 2, Figure S18).

Supplementary Table 2: Comparison of reanalysis to published results

	Reported [6]	Re-analysis	Common
Precursors	40304	52529	
Peptides	35013	41834	33537
Proteins	4984	4703	4566
Proteins detected in > 80%	4077	4262	3979
Median proteins per file	4548	4474	
Median precursors per file	31866	34357	
Proteins with >1 peptide	3985	4275	
Peptide per protein	8.1	9.69	
Inter-site CV unnormalized	57.6	57.2	
Intra-day CV normalized	8.3 ± 16.2	9.25 ± 14.5	
Inter-day CV normalized	11.9 ± 17.2	9.16 ± 13.1	

Inter-site CV normalized	22.0 ± 17.4	21.8 ± 14.4	

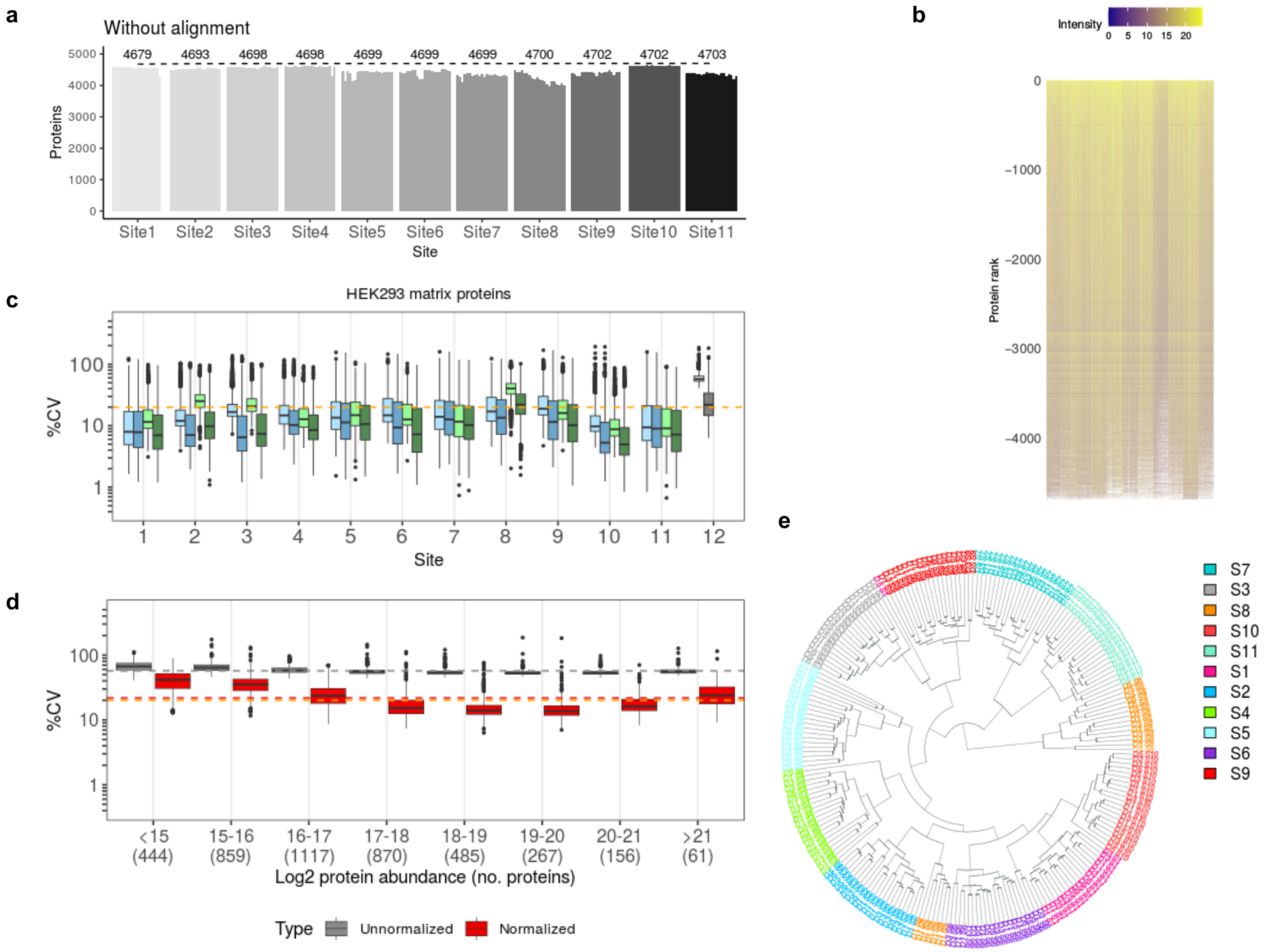

Figure S18. Recreation of published figures. a) The number of proteins detected in each of the 229 SWATH-MS analyses is shown ordered by site of data collection and then chronologically by time of acquisition. After filtering the data set in a global fashion at 1% FDR at the peptide query and protein levels, a protein was considered detected in a given sample when a peak group for that protein was detected at 1% FDR with experiment-wide context. b) A protein abundance matrix on the log2 scale is shown for 229 SWATH-MS analyses from all sites corresponding to the set of proteins shown in a. White indicates a missing protein abundance value where a given protein was not confidently detected in a given sample. The proteins are ordered from top to bottom first by row completeness and then by protein abundance. c) The CV of protein abundances for the 4262 proteins that were detected in >80% all samples were computed at the intra-day level within the site, inter-day with site, and inter-site (i.e., all 229 samples in the study). d) The inter-site CVs were binned based on log2 protein abundance to visualize the dependence of CV on protein abundance. e) The dendrogram for the 229 samples from all sites resulting from hierarchical clustering based on the log2 protein abundances generated from the SWATH-MS data is shown. The sites are color coded as per the legend. The “D” and “S” notation refers to the day and sample number respectively.

4. DIAlignR

To parallelize the alignment, peptides were divided into 10 fractions. Default parameters were obtained using *paramsDIAlignR()*. Parameters *transitionIntensity* and *hardConstrain* were set to True, *globalAlignmentFdr* set to 1e-04, *polyOrd* set to 4, and *RSEdistFactor* set to 4. We explored *mscore* cutoff [1e-04 1.0] by setting *maxFdrQuery*, *alignedFDR1*, and *alignedFDR2* to the cut-off value.

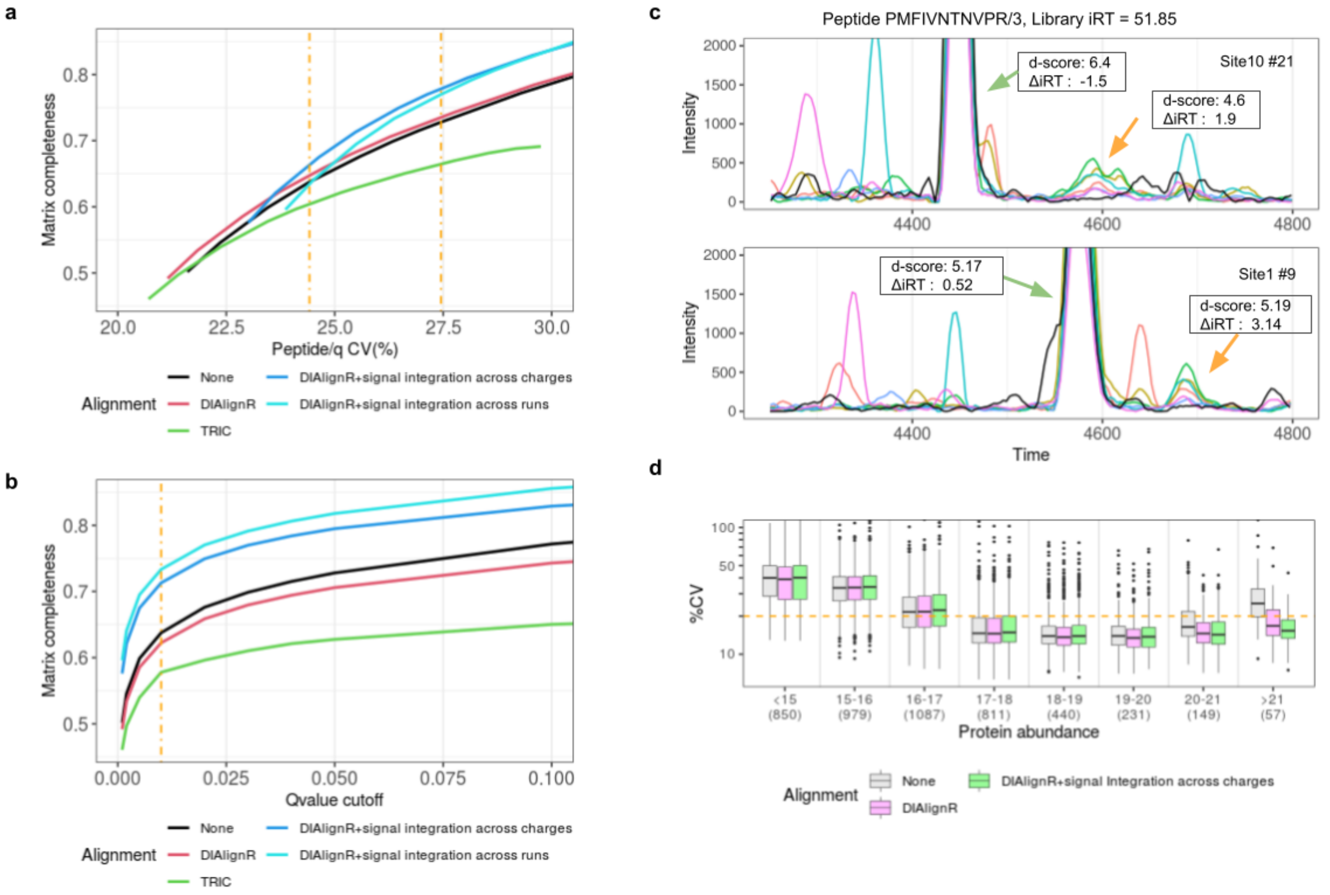

Figure S19. Comparison of signal alignment by DIAlignR to TRIC. Analytes quantified in at least 50% of runs are considered. a,b) Precursor level analysis. For visual clarity only *qvalue* ≥ 0.001 are considered. a) Effect of precursor matrix completeness on CV. b) Effect of PyProphet *qvalue* on matrix completeness is depicted without and with alignment. c) A zoomed-in version of Figure 3c. Black curve is the library MS1 signal. d) CV of proteins is depicted with respect to their mass-spec intensity. Protein intensity is calculated by summing top3 peptides and top5 fragment-ions for a peptide intensity.

5. Across Sites alignment

For peptides, top six fragment-ions are used, selected without alignment. Protein quantification is done using top 3 peptides and their top 5 fragment-ions. The effect of alignment is visible for high intense peptides as wrong intensity would adversely affect the CV. The signal alignment has two modes of action: 1) Select from available scored peaks 2) Create a new peak if no scored peak is found. We

found that action varies with peptide intensity (Figure S20). For high intensity peptides, mostly there is a scored-peak available that is picked by the alignment. On the contrary, for the low intensity peptides, scored-peaks are unavailable within the aligned retention time window, hence, it creates a new peak.

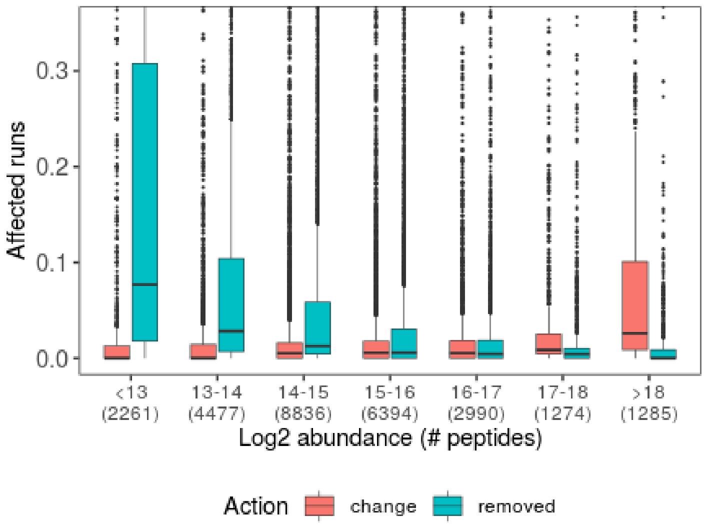

Figure S20. Peak selection after signal alignment. Red box indicates runs for which an already present scored-peak (peak_group_rank ≠ 1) was selected. Blue boxes indicate that scored-peak was not found, hence, a new peak was created. Aligned peaks with peak_group_rank = 1 are excluded from the figure.

6. Comparison of multi-run alignment methods

For multi-run comparison, *RSEdistFactor* was set to 3 and *maxFdrQuery*, *alignedFDR1*, and *alignedFDR2* were set to 0.05. The results were filtered with 1% *mscore* cutoff. For site-specific CV calculation, within-site alignment is performed for each site.

In progressive approach, the master node has more features than the parent runs as *mscore* is set to the minimum of the parent's score. This leads to accumulation of small retention time deviations (Figure S21a) as the tree is traversed to the root. The increment in the RSEs shifts hybrid alignment towards the local alignment, diminishing the benefit of global constraining. Across site, the RSE increases to 100 sec which leads to an adaptive retention time window of 300 sec for hybrid alignment. Given the extracted-ion chromatogram itself is 900 sec, the alignment is likely to behave as local alignment for most of the chromatogram. Nonetheless, the advantage of progressive method for site-specific alignment is that it generates a template chromatogram for a peptide which could be used to curate a chromatogram library [13]. Across sites, since master runs are more distant, the signal around the peak may not be consistent and merging chromatograms may result in copies of the peak in the template. Given the above comparison of Star, MST and Progressive alignment approaches, we are providing a recommendation table (Supplementary Table 5) for running DIAlignR in an experiment.

Supplementary Table 3: CV of precursors at 1% FDR and quantified in all runs.

Multirun method	Number of precursors	CV (median ± sd) %	
		Cross-site (229 runs)	Site-specific (11 sites)
None	7093	18.5 ± 10.2	8.34 ± 11.1
DIAlignR	6057	17.7 ± 6.73	8.04 ± 7.3
DIAlignR + signal integration across charges	8509	19.8 ± 14.3	9.37 ± 10.0

Supplementary Table 4: Number of global alignments calculated

**# Global alignments**	Site-specific	Cross-site
Progressive*	430	26
Star	4540	47672
MST	434	22

* Two runs were clustered outside of the site.

Supplementary Table 5: Comparison of multirun alignment methods

**Multirun Alignment**	**Star-tree**	**Minimum Spanning tree**	**Progressive**
Reference-free	No	No	Yes
Tree building	-	# IDs in each run	# IDs in each run
Order of Global alignment	$O(N^2)$	$O(N)$	$O(N)$
Execution time	Low	Lower	High(merged run)
Single-column alignment	Lesser preferred	Preferred	Preferred
Multi-column alignment	Lesser preferred	Preferred	Not preferred
Disk space requirement	Low	Low	High(merged run)
RAM requirement	High(global align)	Low	Low
Consensus chromatogram	No	No	Yes

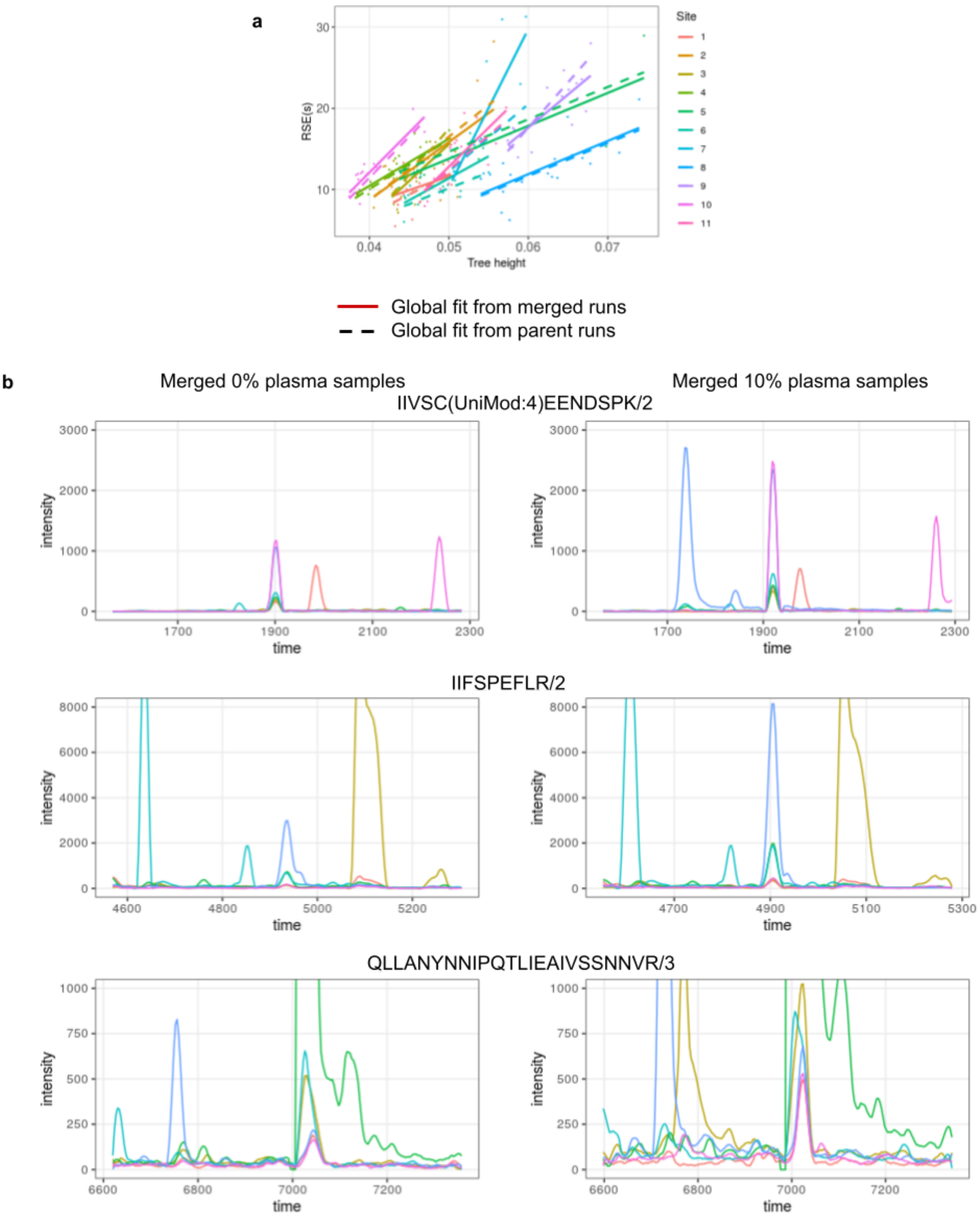

Figure S21. a) Average RSE of children runs for each merged run from progressive alignment. Average RSE of corresponding leaf runs. b) Merged chromatograms of top 3 peptides of *hasB* protein from 11 runs of *S. Pyogenes* cell lysate. Left part depicts merged-chromatograms from 0% plasma samples, whereas, right one has chromatograms from 10% plasma samples.

Supplementary Note 7: *S. Pyogenes* growth in plasma - differential proteomics analysis

The advantage of alignment is to increase the quantitation events while tightly controlling the error-rate. We present here how increasing quantitation events affect the number of peptides and proteins that are differentially expressed.

1. MSConvert + OpenSWATH + PyProphet

The 16 wiff files were converted to mzML using MSConvert without peak-picking. For OpenSWATH following values are used: min_upper_edge_dist = 1, MS2 extraction window = 75 ppm, MS1 extraction window = 35 ppm, DIA extraction window = 75 ppm, extra RT window = 100s, Quadratic regression for ppm mass correction, background subtraction with vertical_division_min, mutual information and MS1 scoring were added. Lossy compression was set to False for converting chrom.mzML to chrom.sqMass files. For PyProphet score XGBoost classifier was used with ms1ms2 level and 0.1 value set for initial FDR.

2. DIAlignR

Run hroest_K120808_Strep10%PlasmaBiolRepl2_R02 was excluded from the signal alignment. Remaining 15 runs were aligned with progressive alignment. Default parameters were obtained using *paramsDIAlignR()*. Parameters *transitionIntensity* and *hardConstrain* were set to True, *globalAlignmentFdr* set to 1e-04, and *RSEdistFactor* set to 4. The alignment cut-offs *maxFdrQuery*, *alignedFDR1*, and *alignedFDR2* were set to 5%. Dynamic programming related factors were set as *goFactor*=1 and *geFactor*=100 and *gapQuantile*=0.8. The final matrix was filtered with *mscore* ≤ 0.025 after signal integration across runs with *qvalue* control. The intensities were median-normalized and log2 transformed, and technical replicates labeled as R01 were discarded from downstream analysis. For the remaining 11 samples, alignment increased the matrix completeness from 58% to 65% (Figure S22). DIAlignR also picks the correct peaks as visible for the most intense ions in the figure.

The data is available at PeptideAtlas repository PASS01508.

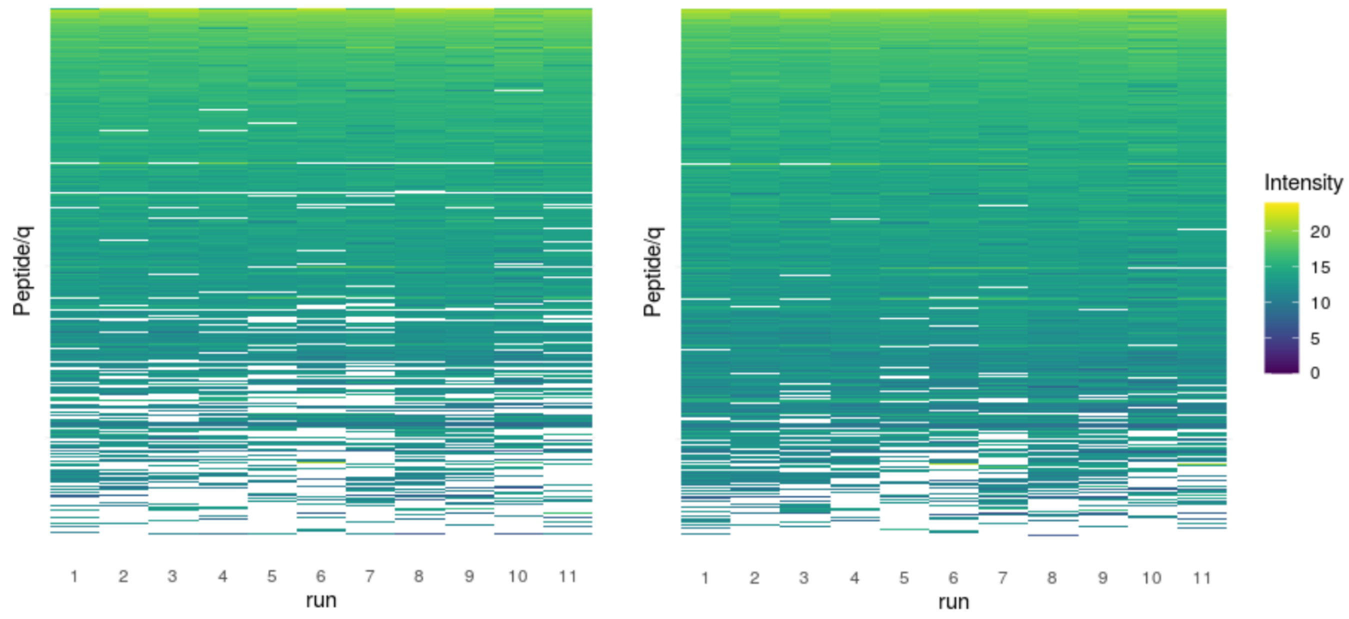

a) Before alignment b) After alignment

Figure S22: A quantification matrix from the SWATH-MS data. The ions are ordered as per their mean intensity. Missingness increases as the intensity reduces. a) Quantification matrix with XGBoost scoring only, b) Matrix with XGBoost scoring followed by progressive alignment.

3. Differential expression

There were 67 proteins found significantly associated with bacterial growth in plasma, that is 10% higher than without signal alignment. The increasing number of significant proteins is not due to the higher *mscore* cutoff (Supp Table 6). The proteins that are not called significant after alignment are mostly due to reduced fold-change. However the new proteins (highlighted in yellow) that are called significant are due to lower *p-value* of differential analysis as matrix completeness increased, resulting in more quantification events backing the fold-change. Next, we were wondering if unwittingly alignment action was biased towards significant genes. No such bias from alignment-action was observed that favored only significant genes (Figure S24).

A volcano plot depicting significant genes is presented in Figure S23. With alignment, we are able to call additional virulence factors hasB, which together with hasA is responsible for the production of hyaluronic acid. This is consistent with the fact that both genes are present on the same operon in the genome of *S. pyogenes* (Figure S26).

Supplementary Table 6: Results of differential proteomics analysis

Alignment	*mscore* cutoff	Significant proteins	
		Identified	Intersect (1% unaligned o/p)

None	0.01	60	60
None	0.025	59	54
Progressive + signal Integration across charges	0.025	67	51
Progressive + signal Integration across runs	0.025	68	51

Supplementary Table 7: Fold change and *p-value* of proteins called significant in either before or after alignment. Newly called proteins are highlighted in yellow.

	Protein	Without alignment		With alignment		Description
		log2(FC)	p-value	log2(FC)	p-value	
1	SPy_1155	1.1	8.3e-05	**0.98**	0.0003	VOC domain-containing protein
2	SPy_1892	1.1	0.009	**0.94**	0.013	Hydrolase_4 domain-containing protein
3	SPy_1434	-1.3	0.0003	**-0.93**	4.7e-05	Putative heavy metal-transporting ATPase
4	SPy_1798	1.0	0.0008	**0.87**	0.002	NA
5	SPy_0913	-1.0	0.003	**-0.69**	0.001	Putative ribosomal protein S1-like DNA-binding protein
6	asnA	-1.1	0.0016	**-0.58**	0.07	Aspartate--ammonia ligase
7	SPy_0721	1.0	0.009	**0.45**	0.02	Flavodoxin
8	msmK	-1.0	0.003	**-0.43**	0.03	Multiple sugar-binding ABC transport system (ATP-binding protein)
9	SPy_0722	-1.1	0.001	**-0.35**	0.13	Chorismate mutase domain-containing protein
10	mutM	2.13	0.01	2.45	**0.0039**	Formamidopyrimidine-DNA glycosylase
11	arsC	0.93	0.1	2.39	**0.0024**	Putative arsenate reductase
12	SPy_1581	0.08	0.80	**1.98**	**0.0098**	Cupin_2 domain-containing protein
13	SPy_0604	1.56	0.012	1.93	**0.0036**	DUF4430 domain-containing protein
14	SPy_0339	-3.68	0.11	-1.91	**0.011**	DnaB_2 domain-containing protein
15	SPy_1134	1.3	0.015	1.85	**0.0017**	Putative ABC transporter (Binding protein)
16	pcrA	-0.43	0.42	1.77	**0.0003**	ATP-dependent DNA helicase
17	recU	1.46	0.012	1.46	**0.0118**	Holliday junction resolvase RecU
18	rpsI	0.67	0.20	**1.26**	**0.0024**	30S ribosomal protein S9
19	rpsT	0.45	0.092	**1.198**	**0.007**	30S ribosomal protein S20
20	SPy_1565	0.96	0.0008	**1.22**	2.4e-05	NA
21	SPy_1691	0.92	0.0015	**1.194**	0.00015	NA
22	ligA	0.89	0.041	**1.149**	**0.008**	DNA ligase
23	SPy_0560	-0.99	0.016	**-1.147**	**0.0081**	ATP-grasp domain-containing protein
24	trmD	-0.39	0.152	**-1.133**	**0.0008**	tRNA (guanine-N(1)-)-methyltransferase
25	hasB	0.93	0.0006	**1.032**	**0.0001**	UDP-glucose 6-dehydrogenase
26	SPy_1344	0.97	0.005	**1.011**	0.0015	(3R)-hydroxymyristoyl-[acyl-carrier-protein] dehydratase

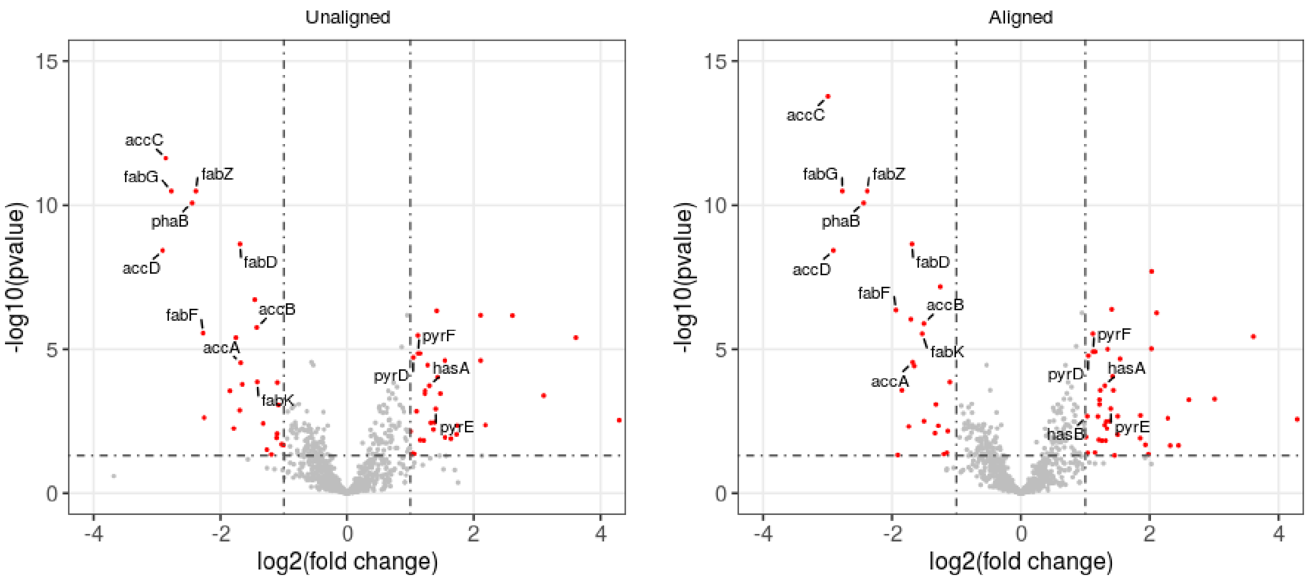

a) Before alignment b) After alignment

Figure S23: Volcano plot depicting significant proteins (red dots). Analysis on a quantitation matrix a) without alignment, and b) after the alignment. Significant genes associated with fatty acid metabolism, pyrimidine biosynthesis and few virulence factors are labelled.

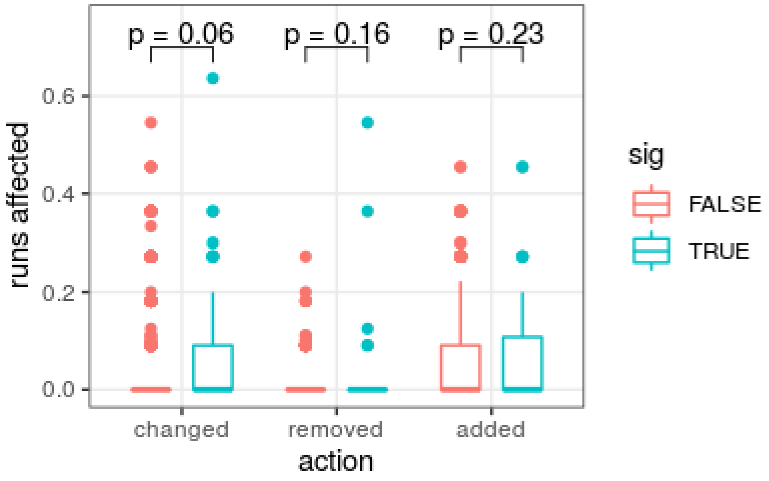

Figure S24: Actions by DIAlignR with respect to significant and non-significant proteins in *S. Pyogenes* cell lysate differential proteome analysis.

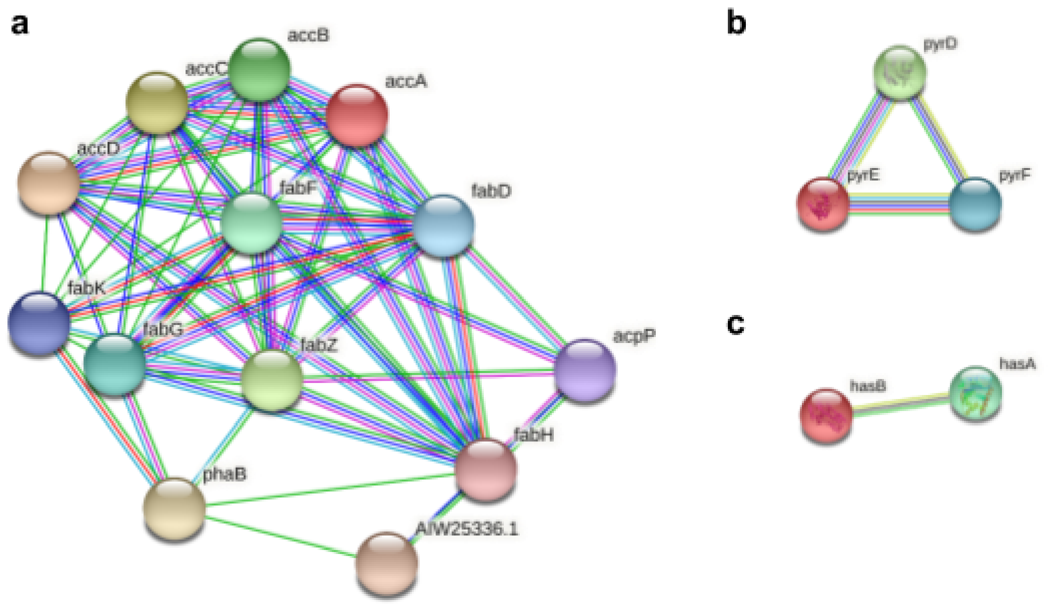

Figure S25: The connected protein networks are fetched from STRING 11.5 [19]. The edges represent protein-protein associations. The edge color indicates the source of interaction available on the STRING db website. a) Fatty Acid Biosynthesis from Local Network Clustering. b) Interactions among the pyrD, pyE, and pyrE proteins found significant. c) Interactions among the virulence factors hasA (Hyaluronan synthase), hasB (UDP-glucose dehydrogenase) found significant.

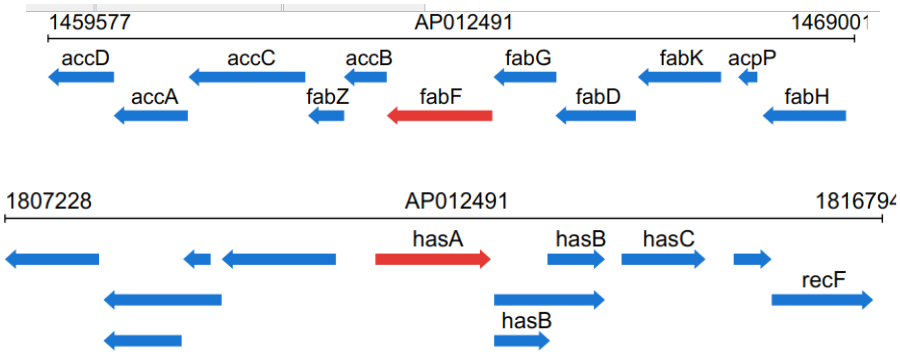

Figure S26: The Genomic locus of *S. Pyogenes* depicting a) FAB proteins and b) virulence factors.

The other important pathways are fatty acid biosynthesis (FAB) and pyrimidine biosynthesis that are found significant in both before and after the alignment (Figure S23). The fold change using all quantified peptides is depicted in Figure S27 for both pathways, and virulence factors.

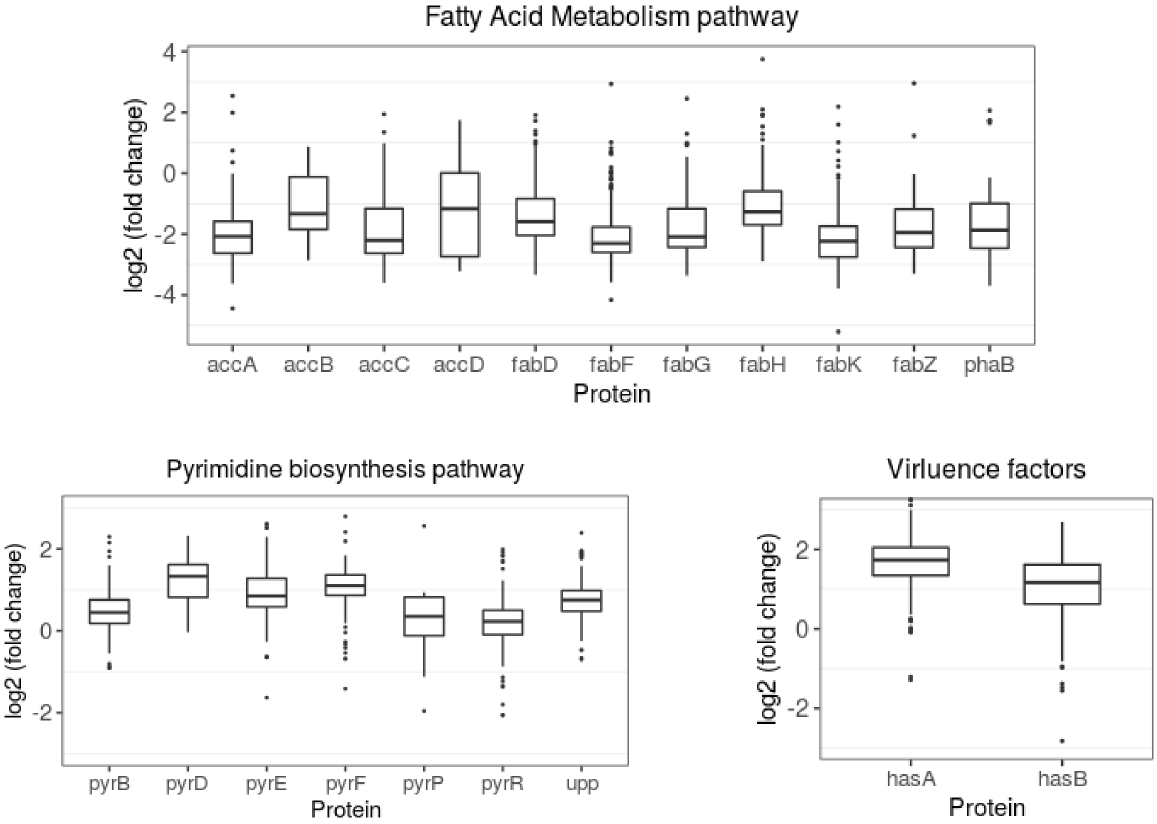

Figure S27: Fold change using all quantified peptides for (top) fatty acid metabolism proteins, (bottom-left) pyrimidine biosynthesis pathway proteins, and (bottom-right) virulence factors.

4. Chromatogram visualization

One advantage of merging chromatograms in progressive alignment is to have a single chromatogram from all runs. This single snap-shot can be used for visually confirming the peak. While merging, each chromatogram is weighed by its *p-value*, hence, the merged chromatogram is a weighted-average of all underlying chromatograms. However, in some cases, it may easily display the differential abundances, as shown for *hasB* protein in Figure S21b.

Supplementary Note 8: Prediabetic study - 949 human plasma runs

We have reanalyzed the data from the integrative personalized omics profiling (iPOP) study.In this study, samples were collected quarterly for 8 years (median 2.8 years). The analysis included plasma proteome as one of the emerging tests for clinics. The cohort comprised 55 women, 52 men with mean age of 53.4 ± 9.2. Based on the steady state plasma glucose (SSPG) level from the insulin suppression test, 35 individuals were classified as insulin resistant (SSPG ≥ 150 mg/dl), 31 individuals as insulin sensitive (SSPG < 150 mg/dl). The status of other 41 individuals was not known as insulin suppression tests were not performed on them. Samples were generally taken every three months

when participants were self-reported as healthy. In total, 576 healthy baselines were profiled, with each participant having 1–34 healthy visits during the study. Additional visits during periods of environmental or medical stress included events of respiratory viral infection (RVI; 54 episodes in 32 participants with a total of 149 visits) with dense sampling in the early phase (two time points during days 1–6), a later phase (day 7–14) and the recovery phase (at weeks 3 and 5). Samples were also taken when other stresses occurred, such as weight gain, antibiotic treatment, colonoscopy, travel and other self-reported acute severe stresses, but these were less frequent.

1. MSConvert + OpenSWATH + PyProphet

The data is already in the mzML format. For OpenSWATH following values are used: min_upper_edge_dist = 1 Da, MS2 extraction window = 67 ppm, MS1 extraction window = 35 ppm, DIA extraction window = 67 ppm, extra RT window = 50s, Quadratic regression for ppm mass correction, background subtraction with vertical_division_min, mutual information and MS1 scoring were added. Lossy compression was set to False for converting chrom.mzML to chrom.sqMass files. For PyProphet score, XGBoost classifier was used with ms1ms2 level, var_library_rootmeansquare as main score and pi0_lambda in the range [0.05 0.99] with step of 0.02. At 1% peptide FDR, we quantified 7297 peptides mapping to 414 proteins (7% protein FDR).

2. DIAlignR

To parallelize the alignment across 949 runs, peptides were divided into 10 fractions. Default parameters were obtained using *paramsDIAlignR()*. Parameters *transitionIntensity* and *hardConstrain* were set to True, *maxFdrQuery*, *alignedFDR1*, and *alignedFDR2* were set to 0.05. Minimum spanning Tree was selected for multirun alignment. The final data matrix was filtered with *mscore* ≤ 0.025 and *qvalue* ≤ 0.025 with signal integration across charges enabled.

3. Insulin resistant v/s insulin sensitive

Statistics for proteins found significant before and after alignment is described in the table below.

Supplementary Table 8: Fold change and *p-value* of proteins.

	Protein	Without DIAlignR		With DIAlignR		**Description**
		log2(FC)	p-value	log2(FC)	p-value	
1	ADIPOQ	0.57	4.38e-12	0.52	6.36e-11	Adiponectin
2	CNDP1	0.33	5.17e-08	0.33	9.76e-08	Beta-Ala-His dipeptidase
3	LPA	0.92	5.67e-08	0.7	5.72e-07	Lipoprotein(a)
4	APOD	0.32	4.35e-06	0.34	4.91e-07	Apolipoprotein D
5	HPR	0.28	6e-04	**0.33**	1.16e-04	Haptoglobin-related protein
6	IGHD	0.53	5.65e-03	0.61	4.09e-03	Immunoglobulin heavy constant delta
7	IGLV6-57	-0.55	6.58e-06	-0.56	3.26e-07	Immunoglobulin lambda variable 6-57
8	IGKC	-0.34	0.027	-0.50	**5.92e-05**	Immunoglobulin kappa constant

9	HP	-0.3	0.102	-0.5	**6.53e-05**	Haptoglobin
10	IGHG2	-0.31	0.015	-0.41	**2.97e-04**	Immunoglobulin heavy constant gamma 2
11	PZP	0.43	6.09e-03	0.46	4.3e-03	Pregnancy zone protein

As the *mscore* cutoff is increased, the unaligned data has fewer missing values, however the incorporation of false peaks affect the significant estimation in differential analysis. As demonstrated in Suppl Table 7, increasing *mscore* leads to fewer proteins being associated with IR. However, since alignment reduces error-rate, increasing *mscore* cutoff to 2.5% results in a consistent and higher number of proteins. Increasing it further to 5% and 10% level, fewer known genes are found to be significant. Proteins APOC4, IGHG4 (at 5% cutoff) have the backing of few literatures. Proteins MAP7D3 and NPHP3, found at 10% cutoff have no known evidence of association with insulin sensitivity, hence are questions. In both cases, we lose a known biomarker HPR from the analysis.

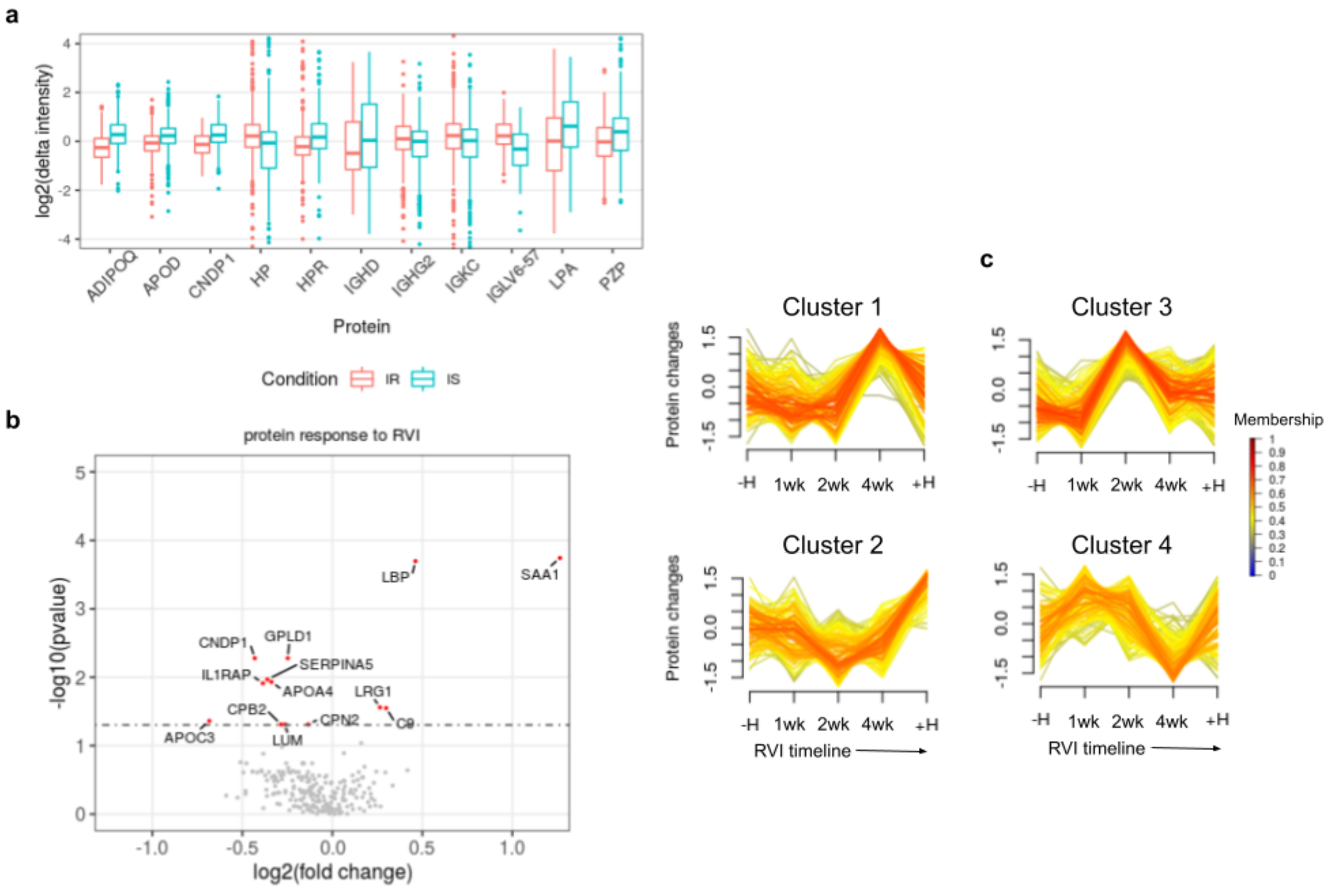

Figure S28 a) Distribution of peptide intensities of significant proteins for both insulin resistant (IR) and insulin sensitive (IS) after correcting for batch effects, acquisition order and participant-specific effects. b) Volcano plot depicting proteins that changes significantly during RVI compared to baseline samples of an unaligned dataset. c) Cluster 1 and Cluster 2 from the unaligned dataset. The remaining Cluster 3 and Cluster 4 are also depicted.

Supplementary Table 9: Effect of *mscore* (FDR) control on IR associated proteins

FDR(%)	Before alignment	After alignment	
		Signal Integration	Gene names
1	ADIPOQ, CNDP1, LPA, APOD, IGHD, IGLV6-57, PZP		
2.5	ADIPOQ, IGLV6-57, APOD, LPA, CNDP1, PZP	Across charges	ADIPOQ, CNDP1, LPA, APOD, HPR, IGHD,IGLV6-57,IGKC,HP, IGHG2,PZP
2.5		Across runs	ADIPOQ,CNDP1,LPA,APOD,HPR,HP, IGHD, IGLV6-57, IGKC, PZP, IGHG2
5	ADIPOQ, IGLV6-57, APOD, LPA, PZP	Across charges	ADIPOQ, LPA, APOD, IGLV6-57, IGKC, HP, IGHG2, PZP
5		Across runs	ADIPOQ, LPA, APOD, IGLV6-57, PZP, IGKC, HP, IGHG2, **APOC4, IGHG4**
10		Across charges	ADIPOQ, LPA, APOD, IGHG4, IGLV6-57, IGKC, APOC4, **MAP7D3**, HP, IGHG2, **NPHP3**
10		Across runs	ADIPOQ, LPA, APOD, IGHG4, IGLV6-57, IGKC, APOC4, **MAP7D3**, HP, IGHG2, **NPHP3**

4. Change in proteome during respiratory viral infection

Healthy visits that occurred within 180 days of infection are kept for this analysis. Infection period is categorized into five events:

-H: Healthy before infection

IE: Infection Early (1-14 days after infection)

IL: Infection Late (14-21 days after infection)

IR: Infection Recovery (3-4 weeks after infection)

+H: Healthy time points (4 weeks after infection)

We detect 13 proteins to be statistically significant (Figure 5c) with alignment compared to eight proteins found from the unaligned data, as described in Suppl Table 8,9. To identify the function of these proteins, we used the IMPaLA tool [23] pathway over-representation analysis without background list. Two proteins GC and ITIH3 were not found significant in the aligned data due to DIAlignR picking different peaks. GC gene encodes vitamin D binding protein (DBP) that has been implicated in the central nervous system and hepatitis C viral infection. There are also some initial reports establishing COVID-19 prevalence and mortality to rs7041 locus of DBP. For the other gene ITIH3, there is limited information available. It is speculated that its product protein may act as a carrier of hyaluronan in serum which is believed to play a role in virulence. Nonetheless, the new significant proteins are due to

lower *p-value* of differential analysis as with alignment correct peaks are picked/quantified (Figure S29), as observed for *S. Pyogenes* analysis as well.

Supplementary Table 10: *p-value* of significant proteins from RVI samples with DIAlignR

	Protein	p-value	Description
1	**CPN2**	2.8e-03	Carboxypeptidase N subunit 2
2	**LUM**	2.72e-03	Lumican
3	**CPB2**	2.65e-03	Carboxypeptidase B2
4	**APOC3**	1.92e-03	Apolipoprotein C-III
5	**C9**	1.12e-03	Complement component C9
6	LRG1	9.73e-04	Leucine-rich alpha-2-glycoprotein
7	**IL1RAP**	3.79e-04	Interleukin-1 receptor accessory protein
8	APOA4	3.1e-04	Apolipoprotein A-IV
9	**SERPINA5**	2.37e-04	Plasma serine protease inhibitor
10	GPLD1	9.29e-05	Phosphatidylinositol-glycan-specific phospholipase D
11	CNDP1	9.24e-05	Beta-Ala-His dipeptidase
12	LBP	1.77e-06	Lipopolysaccharide-binding protein
13	SAA1	7.97e-07	Serum amyloid A-1 protein

Supplementary Table 11: *p-value* of significant proteins from RVI samples without DIAlignR

	Protein	p-value	Description
1	LRG1	9.73e-04	Leucine-rich alpha-2-glycoprotein
2	**ITIH3**	5.09e-04	Inter-Alpha-Trypsin Inhibitor Heavy Chain 3
3	**GC**	2.84e-04	Vitamin D-binding protein
4	APOA4	2.47e-04	Apolipoprotein A-IV
5	GPLD1	1.06e-04	Phosphatidylinositol-glycan-specific phospholipase D
6	CNDP1	8.83e-06	Beta-Ala-His dipeptidase
7	LBP	4.44e-06	Lipopolysaccharide-binding protein
8	SAA1	9.8e-07	Serum amyloid A-1 protein

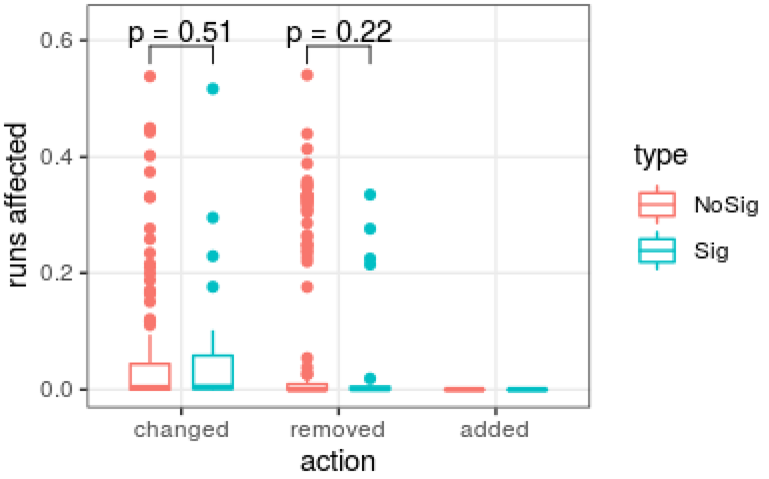

Figure S29. Actions by DIAlignR with respect to significant and non-significant proteins in clinical plasma data analysis.

The core-proteins for each cluster from fuzzy c-means clustering are presented in tables below. Proteins involved in the mentioned pathway are in boldface.

Supplementary Table 12: Core genes in each cluster (with alignment)

Cluster 1	Cluster 2	Cluster 3	Cluster 4
Adaptive Immune System (R-HSA-1280218)	FCGR activation (R-HSA-2029481)	Complement and coagulation cascades (WP558)	Metabolism of proteins (R-HSA-392499)
**C3**<br>**IGKV1D-33**<br>**IGLV3-25**<br>**IGHD**<br>APOC3<br>ORM1<br>PROC<br>F13B<br>CLEC3B<br>DBH<br>C4BPB<br>MST1<br>SELENOP<br>**IGLV3-21**<br>PI16<br>PRG4	**IGKV1D-16**<br>**IGLV3-19**<br>**IGHV3-13**<br>**IGHV3-53**<br>**IGHV3-7**<br>**IGHV4-39**<br>**IGKC**<br>**IGHG4**<br>APOC2<br>GP1BA<br>THBS1<br>LPA<br>CETP<br>F5<br>AZGP1<br>BTD<br>INHBC<br>HBA2<br>CNDP1<br>SERPINA10	CP<br>**F9**<br>PLG<br>**F12**<br>KNG1<br>IGHG3<br>RBP4<br>TF<br>**KLKB1**<br>C4BPA<br>C8G<br>CLU<br>ITIH2<br>AFM<br>HGFAC<br>ADIPOQ	CFB<br>**SERPINC1**<br>**APOA2**<br>**FGA**<br>FGB<br>**APCS**<br>APOH<br>TTR<br>**ALB**<br>GC<br>**APOB**<br>HRG<br>SERPING1<br>**BCHE**<br>PZP<br>CFHR2<br>NPHP3<br>RBFA<br>**CPB2**<br>FETUB

Supplementary Table 13: Core genes in each cluster (without alignment)

Cluster 1	Cluster 2	Cluster 3	Cluster 4
Adaptive immune system (R-HSA-1280218)	No pathway	Complement and coagulation cascades (WP558)	Metabolism (R-HSA-1430728)
**C3**<br>**IGHV1-2**<br>**IGLV1-40**<br>**IGHV1-46**<br>**IGHV4-39**<br>**IGHM**<br>F10<br>JCHAIN<br>RBP4<br>ORM1<br>PROC<br>F13B	IGKV3-15<br>IGKC<br>PPBP<br>CFH<br>ITIH2<br>GPX3<br>VARS1<br>IGFALS<br>LUM<br>GPLD1	**SERPING1**<br>**C8G**<br>**CLU**<br>**F9**<br>**KNG1**<br>IGHG1<br>A1BG<br>IGLL5<br>CPN2	**PON1**<br>**TTR**<br>**BCHE**<br>**APOA2**<br>**APOC3**<br>**APOB**<br>**APOA4**<br>**ALB**<br>**GC**<br>HP<br>PLG<br>F12

ITIH1<br>C4BPB<br>SELENOP<br>APOC4<br>PLTP<br>LGALS3BP<br>APOF<br>MYBPC2<br>FCN2<br>CPB2<br>INPP5E<br>C1RL<br>FCGBP			CFB<br>IGHA1<br>FGA<br>FGB<br>FGG<br>APCS<br>APOH<br>AHSG<br>IGKV3-11<br>PZP<br>AZGP1<br>AFM<br>ITIH4<br>NPHP3<br>SLFN11

5. Comparison with original paper

The six proteins reported to be associated with Insulin resistance are ADIPOQ, MCAM, APOD, PLTP, APOC4 and VTN. We do find ADIPOQ and APOD in our analysis, MCAM protein was filtered out as it was not identified in >40% runs, other three proteins PLTP, APOC4 and VTN did have p-value less than 0.05, however, the effect-size was not higher than log2(1.25). Beside using older versions of proteomics data analysis software and not performing retention time alignment across all runs, analysis methodology is one of the main factors. The study was focused on combining and analyzing multi-omics data, hence, it is possible that due to including so many hypotheses, the other proteins were missed. In addition, the analysis was performed with protein-level intensities and missing values were imputed compared to this paper where peptide-level intensities are followed without any imputation. Moreover, the association was determined to SSPG level in the original study, whereas, we have performed binary classification for being either insulin resistance or insulin sensitive.

Supplementary Note 9: Software Versions

Although DIAlignR uses raw chromatogram data compared to features used by TRIC, the method is scalable to 1000s of runs due to the embarrassingly parallel nature of alignment across peptides. Hence, the computation can be divided across multiple CPUs reducing memory requirements and execution time. The table below shows the computing cost comparison of both tools.

Supplementary Table 14: Computational cost for TRIC and DIAlignR

Study	# Peptides	Cost	DIAlignR	TRIC
Multilab study: 229 HEK293 cell lysate runs	41834	RAM/cpu	10G	24 G
		Time/cpu	4 hr	2 hr
		cpus	10	1

Study	# Peptides	Cost	DIAlignR	TRIC
Prediabetic study: 949 plasma runs	11419	RAM/cpu	12G	96 G
		Time/cpu	2.5 hr	20 hr
		cpus	10	1

References

1. Gupta S, Ahadi S, Zhou W, Röst H. DIAlignR provides precise retention time alignment across distant runs in DIA and targeted proteomics. *Mol. Cell. Proteomics* **18**, 806-817 (2019).
2. Gupta S, Röst H. Automated Workflow For Peptide-level Quantitation from DIA/SWATH-MS Data. *Methods in Molecular Biology* (2020).
3. Gupta S, Sing J, Mahmoodi A, Röst H. DrawAlignR: An interactive tool for across run chromatogram alignment visualization. *Proteomics* (2020).
4. Röst, H.L. *et al.* OpenSWATH enables automated, targeted analysis of data-independent acquisition MS data. *Nature Biotechnology* **32**, 219–223 (2014).
5. Röst, H. L. *et al.* TRIC: an automated alignment strategy for reproducible protein quantification in targeted proteomics. *Nature Methods* **13**, 777–783 (2016).
6. Collins B.C. *et al.* Multi-laboratory assessment of reproducibility, qualitative and quantitative performance of SWATH-mass spectrometry. *Nat Commun* **8**(1), 291 (2017).
7. Zhou, W. *et al.* Longitudinal multi-omics of host–microbe dynamics in prediabetes. *Nature* **569**, 663–671 (2019).
8. Bichmann L, Gupta S, Rosenberger G, Kuchenbecker L, Sachsenberg T, Ewels P, Alka O, Pfeuffer J, Kohlbacher O, Röst H. DIAproteomics: A Multifunctional Data Analysis Pipeline for Data-Independent Acquisition Proteomics and Peptidomics. *J. Proteome Res.* **7**, 3758–3766 (2021).
9. Gillet, L. C. *et al.* Targeted data extraction of the MS/MS spectra generated by data-independent acquisition: a new concept for consistent and accurate proteome analysis. *Mol. Cell. Proteomics* **11**, (2012).
10. Wu, L., Amon, S., and Lam, H. A hybrid retention time alignment algorithm for SWATH-MS data. *Proteomics* **16**, 2272–2283 (2016).
11. Li, Y., *et al.* Group-DIA: analyzing multiple data-independent acquisition mass spectrometry data files. *Nat Methods* **12**, 1105–1106 (2015).
12. Escher, C. *et al*. Using iRT, a normalized retention time for more targeted measurement of peptides. *Proteomics* **12**,1111–1121 (2012).
13. Searle, B.C., Pino, L.K. *et al*. Chromatogram libraries improve peptide detection and quantification by data independent acquisition mass spectrometry. *Nat Commun* **9**, 5128 (2018).
14. Rosenberger, G., Bludau, I., Schmitt, U. *et al.* Statistical control of peptide and protein error rates in large-scale targeted data-independent acquisition analyses. *Nat Methods* **14**, 921–927 (2017).
15. Demichev, V., Messner, C.B., Vernardis, S.I. *et al.* DIA-NN: neural networks and interference correction enable deep proteome coverage in high throughput. *Nat Methods* **17**, 41–44 (2020).
16. W. Hendrix. *et al.* "A scalable algorithm for single-linkage hierarchical clustering on distributed-memory architectures," *IEEE Symposium on Large-Scale Data Analysis and Visualization (LDAV)*, 7-13 (2013).
17. Rosenberger, G. *et al.* A repository of assays to quantify 10,000 human proteins by SWATH-MS. *Sci. Data* **1**, 140031 (2014).
18. JJ, Davis. *et al.* The PATRIC Bioinformatics Resource Center: expanding data and analysis capabilities. *Nucleic Acids Res*, **48**(D1):D606-D612 (2020).
19. Szklarczyk, D. *et al.* STRING v11: protein-protein association networks with increased coverage, supporting functional discovery in genome-wide experimental datasets. *Nucleic Acids Res*. 47(D1):D607-D613 (2019).

20. Čuklina J. *et al.* Diagnostics and correction of batch effects in large-scale proteomic studies: a tutorial. *Mol Syst Biol*. **17**: e10240 (2021).
21. Bruderer R. *et al.* Analysis of 1508 Plasma Samples by Capillary-Flow Data-Independent Acquisition Profiles Proteomics of Weight Loss and Maintenance. *Mol Cell Proteomics*. **6**, 1242-1254 (2019).
22. Liu Y, Buil A, Collins BC, *et al*. Quantitative variability of 342 plasma proteins in a human twin population. *Mol Syst Biol.* 11(1):786 (2015).
23. Kamburov A, Cavill R, Ebbels TM, Herwig R, Keun HC. Integrated pathway-level analysis of transcriptomics and metabolomics data with IMPaLA. *Bioinformatics* **27**: 2917-2918, (2011).
24. Futschik, M. E. & Carlisle, B. Noise-robust soft clustering of gene expression time-course data. *J. Bioinform. Comput. Biol.* **3**, 965–988 (2005).
25. Junker F, Gordon J, Qureshi O. Fc Gamma Receptors and Their Role in Antigen Uptake, Presentation, and T Cell Activation. *Front Immunol*, **11**:1393 (2020).
