# Supplementary material for: Achieving quantitative reproducibility in label-free multisite DIA experiments through multirun alignment
Source: Commun Biol. 2023 Oct 30;6:1101. doi: 10.1038/s42003-023-05437-2 (PMC10616189; doi:10.1038/s42003-023-05437-2)
Supplement: Supplementary file 2 — Description of Additional Supplementary Files [file 42003_2023_5437_MOESM2_ESM.pdf]

Description of Additional Supplementary Files

File name: Supplemental Data
Description: Numerical data for figures.
