# Supplementary material for: Achieving quantitative reproducibility in label-free multisite DIA experiments through multirun alignment
Source: Commun Biol. 2023 Oct 30;6:1101. doi: 10.1038/s42003-023-05437-2 (PMC10616189; doi:10.1038/s42003-023-05437-2)
Supplement: Supplementary file 4 — Reporting Summary [file 42003_2023_5437_MOESM4_ESM.pdf]

nature portfolio

Corresponding author(s): Hannes Röst

Last updated by author(s): Sep 12, 2023

Reporting Summary

Nature Portfolio wishes to improve the reproducibility of the work that we publish. This form provides structure for consistency and transparency in reporting. For further information on Nature Portfolio policies, see our Editorial Policies and the Editorial Policy Checklist.

Statistics

For all statistical analyses, confirm that the following items are present in the figure legend, table legend, main text, or Methods section.

n/a	Confirmed	
☐	☒	The exact sample size (*n*) for each experimental group/condition, given as a discrete number and unit of measurement
☒	☐	A statement on whether measurements were taken from distinct samples or whether the same sample was measured repeatedly
☐	☒	The statistical test(s) used AND whether they are one- or two-sided<br>*Only common tests should be described solely by name; describe more complex techniques in the Methods section.*
☐	☒	A description of all covariates tested
☐	☒	A description of any assumptions or corrections, such as tests of normality and adjustment for multiple comparisons
☐	☒	A full description of the statistical parameters including central tendency (e.g. means) or other basic estimates (e.g. regression coefficient) AND variation (e.g. standard deviation) or associated estimates of uncertainty (e.g. confidence intervals)
☐	☒	For null hypothesis testing, the test statistic (e.g. *F*, *t*, *r*) with confidence intervals, effect sizes, degrees of freedom and *P* value noted<br>*Give P values as exact values whenever suitable.*
☒	☐	For Bayesian analysis, information on the choice of priors and Markov chain Monte Carlo settings
☒	☐	For hierarchical and complex designs, identification of the appropriate level for tests and full reporting of outcomes
☒	☐	Estimates of effect sizes (e.g. Cohen's *d*, Pearson's *r*), indicating how they were calculated

Our web collection on statistics for biologists contains articles on many of the points above.

Software and code

Policy information about availability of computer code

Data collection	The code is open-source and available at https://github.com/shubham1637/DIAlignR/
Data analysis	Following software and their docker images are used in the analysis<br>MSConvert chambm/pwiz-skyline-i-agree-to-the-vendor-licenses:3.0.21354-9ee14c7<br>OpenSWATH openms/executables@sha256:84191d62898d<br>PyProphet pyprophet/pyprophet:2.1.10<br>TRIC shubham1637/msproteomicstools:0.11.0<br>The scripts to use these tools is available at https://github.com/shubham1637/DIAlignR/wiki

For manuscripts utilizing custom algorithms or software that are central to the research but not yet described in published literature, software must be made available to editors and reviewers. We strongly encourage code deposition in a community repository (e.g. GitHub). See the Nature Portfolio guidelines for submitting code & software for further information.

Data

Policy information about availability of data

All manuscripts must include a data availability statement. This statement should provide the following information, where applicable:

- Accession codes, unique identifiers, or web links for publicly available datasets
- A description of any restrictions on data availability
- For clinical datasets or third party data, please ensure that the statement adheres to our policy

For manual annotation and analysis of bacterial growth, we have used previously published data from PASS01508. For multi-species analysis, we use data previously published and available from PXD002952. The chromatograms, features and other results of this paper are added in the manualAnnotation and differentialAnalysis directories. Multisite data and clinical plasma dataset were fetched from PXD004886 and the iPOP portal, respectively. The libraries used in their analysis and results are uploaded to Zenodo (zenodo.org/record/6677715).

Research involving human participants, their data, or biological material

Policy information about studies with human participants or human data. See also policy information about sex, gender (identity/presentation), and sexual orientation and race, ethnicity and racism.

Reporting on sex and gender	Sex was not considered a term in the analysis, we used patient Id as a term in the analysis.
Reporting on race, ethnicity, or other socially relevant groupings	We did not use the race, ethnicity and other socially relevant groupings.
Population characteristics	The data is taken from already published study. The cohort comprised 55 women, 52 men with mean age of 53.4 ± 9.2.
Recruitment	The data is taken from already published study.
Ethics oversight	The data is taken from already published study.

Note that full information on the approval of the study protocol must also be provided in the manuscript.

Field-specific reporting

Please select the one below that is the best fit for your research. If you are not sure, read the appropriate sections before making your selection.

☒ Life sciences ☐ Behavioural & social sciences ☐ Ecological, evolutionary & environmental sciences

For a reference copy of the document with all sections, see nature.com/documents/nr-reporting-summary-flat.pdf

Life sciences study design

All studies must disclose on these points even when the disclosure is negative.

Sample size	The data is taken from already published study.
Data exclusions	We only analyzed 925 runs out of 949 samples which exhibited 85% completeness.
Replication	NA
Randomization	NA
Blinding	The data is taken from already published study.

Reporting for specific materials, systems and methods

We require information from authors about some types of materials, experimental systems and methods used in many studies. Here, indicate whether each material, system or method listed is relevant to your study. If you are not sure if a list item applies to your research, read the appropriate section before selecting a response.

Materials & experimental systems

n/a	Involved in the study
☒	☐ Antibodies
☒	☐ Eukaryotic cell lines
☒	☐ Palaeontology and archaeology
☒	☐ Animals and other organisms
☐	☒ Clinical data
☒	☐ Dual use research of concern
☒	☐ Plants

Methods

n/a	Involved in the study
☒	☐ ChIP-seq
☒	☐ Flow cytometry
☒	☐ MRI-based neuroimaging

Clinical data

Policy information about clinical studies

All manuscripts should comply with the ICMJE guidelines for publication of clinical research and a completed CONSORT checklist must be included with all submissions.

Clinical trial registration	The data is taken from already published study.
Study protocol	The data is taken from already published study.
Data collection	The data is taken from already published study.
Outcomes	The data is taken from already published study.
